# Multi-staged mineralization and biomarker preservation in a 113-million-year-old pterosaur bone via redox shifts in diagenesis

**DOI:** 10.1016/j.isci.2026.116199

**Published:** 2026-06-18

**Authors:** Kliti Grice, Stephen F. Poropat, Lorenz Schwark, Maria A. Diaz Mateus, Paul F. Greenwood, Luke M. Brosnan, Madison Tripp, Amy L. Elson, Andrew J.Y. Jian, Antônio A.F. Saraiva, Renan A.M. Bantim, Julien Demore, Alex I. Holman, Michael E. Böttcher, Adele H. Pentland, Robert H.C. Madden, Peter Hopper, Xiao Sun, Aaron Dodd, Arthur V. de Oliveira, Pieter T. Visscher, William D.A. Rickard, Juliana M. Sayão, Hossein Rahimpour-Bonab, Iris Schmiedinger, Victor O. Leshyk, Alexander W.A. Kellner

**Affiliations:** 1 Western Australian Organic and Isotope Geochemistry Centre, Curtin University, Perth, WA, Australia; 2 Institute of Geosciences, Christian-Albrechts-University, Kiel, Schleswig-Holstein, Germany; 3 Department of Biological Sciences, Regional University of Cariri, Crato, Ceará, Brazil; 4 Geochemistry and Isotope Biogeochemistry, Leibniz-Institute for Baltic Sea Research (IOW), Warnemünde, Mecklenburg-Vorpommern, Germany; 5 Marine Geochemistry, University of Greifswald, Greifswald, Mecklenburg-Vorpommern, Germany; 6 Interdisciplinary Faculty, University of Rostock, Rostock, Mecklenburg-Vorpommern, Germany; 7 Microanalysis Australia, Perth, WA, Australia; 8 John de Laeter Centre, Curtin University, Perth, WA, Australia; 9 Department of Marine Sciences, University of Connecticut, Groton, CT, USA; 10 Laboratory of Systematics and Taphonomy of Fossil Vertebrates, Department of Geology and Paleontology, Museu Nacional (MN)/Federal University of Rio de Janeiro (UFRJ), Rio de Janeiro, Brazil; 11 Azolla Biodesign, Sedona, AZ, USA

**Keywords:** Earth sciences, Geology, Methods in earth sciences, Biogeochemistry

## Abstract

The combined preservation of soft tissues, biomineralized structures, and molecular biomarkers is rare; yet, such finds offer key insights into ancient physiology, ecology, and taphonomy. We integrate organic geochemical analyses with high-resolution micro-mineral imaging of a three-dimensionally preserved Cretaceous pterosaur wing phalanx from Brazil to reveal steroid biomarkers and multi-stage mineralization pathways underlying its preservation. A localized redox shift toward acidic, oxidative conditions around the carcass played a central role. Microbial decay generated acidity that promoted early phosphate mineralization (fluorapatite), stabilizing tissues. This fluorapatite is associated with barite and celestite indicating a microenvironment with enhanced microbial sulfate production. Following phosphatization, three phases of carbonate mineralization encapsulated organic compounds, protecting them from diagenetic alteration. Molecular analyses report steroids in pterosaurs, with δ^13^C values indicating a fish- and cephalopod-based diet, highlighting early mineralization as key to long-term biomolecule preservation.

## Highlights

• Rare fossil preserves soft tissue, minerals, and biomarkers in pterosaur bone
• Acidic, oxidative decay drove early mineralization and tissue stabilization
• Multi-stage mineralization protected organic molecules over deep time
• First steroid evidence reveals pterosaur diet of fish and cephalopods

## Graphical abstract

**Figure:**
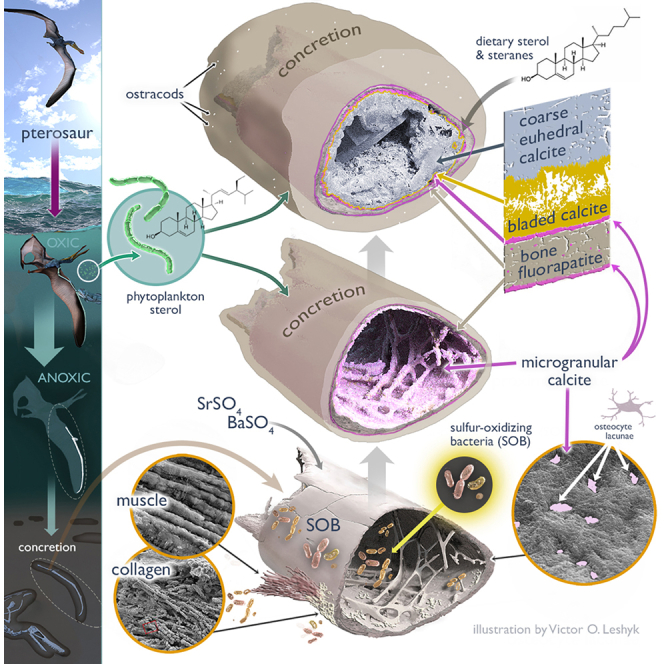

## Introduction

The geological preservation of high-quality fossils typically requires specific geochemical and microbial conditions favorable to the mineralization of biological remains.^1^^,^^2^^,^^3^^,^^4^^,^^5^^,^^6^^,^^7^^,^^8^ Fossil preservation is commonly associated with anoxic or low-oxygen environments, in which destructive aerobic microbes are stymied, and where mineral precipitations (e.g., calcium phosphate) can effectively sequester biological tissues prior to major degradation.^5^ However, this traditional view overlooks some of the potential impacts of a wide range of microorganisms which inhabit anoxic or low-oxygen sediments, sulfate reducing bacteria (SRB) connect the breakdown and transformation of organic matter into carbon dioxide.^9^ Furthermore, microbial sulfur cycling can create localized environments conducive to rapid mineral formation.^10^^,^^11^^,^^12^^,^^13^^,^^14^ Several key studies have shown that anaerobic decay-induced microenvironments trigger the conditions—e.g., redox fluctuations, or more specifically oxidation- reduction potential (ORP)^15^—amenable for phosphatic mineralization of soft tissues.^16^^,^^17^^,^^18^ The aforementioned studies have demonstrated that these processes can produce geochemical conditions and microbiomes supportive of exceptional fossil preservation, although clearly, there is still much to learn about the biotic and abiotic pathways supportive of fossil preservation, particularly those maintained with exceptional form (e.g., three dimensions, 3D).

The Lower Cretaceous Romualdo Formation (Santana Group, Araripe Basin) of Brazil is world-renowned for its exceptionally preserved vertebrate fossils, notably its panoply of pterosaurs.^19^^,^^20^^,^^21^^,^^22^ The best preserved vertebrate fossils from this formation are those encased in calcium carbonate concretions, some of which retain extraordinary anatomical detail, including internal organs, muscle tissue, scaly and filamentous integument, and fine bone structures.^23^^,^^24^^,^^25^^,^^26^^,^^27^^,^^28^^,^^29^^,^^30^

The present study reveals that a pterosaur wing phalanx, three-dimensionally preserved within a carbonate concretion from the Romualdo Formation, was fossilized via a cascading mineralization route. Micro- and macro-scale mineral imaging provides evidence for a more nuanced understanding of how localized redox conditions and the microbiomes they support can facilitate fossil preservation, especially in dominantly sulfur-rich paleoenvironments like the Romualdo Formation (Figure S1).

For further details of the samples, analytical standards and methods employed in this study, please see supplemental information, Figures S1 and S25, and Videos S1.

Video S1. Pterosaur micro-CT data.avi

## Results and discussion

### Depositional environment, geology, and pterosaur bone composition

The Romualdo Formation was deposited in a rift basin that occupied the Araripe Basin of northeast South America during the late Early Cretaceous.^31^^,^^32^^,^^33^ The epeiric seaway that occupied this basin was relatively deep, stratified, and brackish-hypersaline during the temporal window in which concretion formation occurred in anoxic substrates below wave base.^32^ The concretion-bearing horizons correspond to the Aptian-Albian boundary, which is marked worldwide by the Kilian sub-event (Oceanic Anoxic Event 1b).^32^ Bottom waters and the lower part of the photic zone within the water column were euxinic.^31^^,^^33^ The prevalence of calcium carbonate concretions suggests the bottom water was, at the least, neutral to alkaline during their formation window.^34^ While the benthic fauna was depauperate (effectively limited to salinity-tolerant ostracods and foraminiferans), the nektonic fauna was quite diverse, with abundant marine fish remains and rarer fossils of aquatic turtles and crocodyliforms. Terrestrial dinosaurs and volant pterosaurs were also preserved, albeit relatively rarely.^19^^,^^21^^,^^22^

The pterosaur specimen studied herein (Museu de Paleontologia Plácido Cidade Nuvens, Santana do Cariri, Ceará, Brazil [MPSC R] 1395) was collected from the Sítio Baixa Grande locality (Ceará State), in the northwestern region of the Araripe Basin (Figure S1). This specimen comprises a partial left wing that has previously been referred to the clade Anhangueridae.^35^^,^^36^ We focused only on the proximal portion of a wing phalanx (missing the articular surface). Typical of pterosaur long bones, the MPSC R 1395 phalanx is thin-walled and hollow,^37^^,^^38^ with some trabecular struts evident (visualized using micro-CT [μCT] data).^39^ One longitudinal section of the endosteal surface, interpreted here as the leading edge of the wing phalanx, hosts a high density of short struts. Very few struts span the diameter of the medullary cavity, and at least one of these is braced by an auxiliary strut. The lamellar structure (layered arrangement) of the cortical bone is well-preserved, and osteocyte lacunae and vascular canals are common. High magnification reveals canaliculi, which are tiny channels through the bone matrix that allow for the exchange of nutrients between osteocytes (bone cells) and blood vessels.

**Figure 1:**
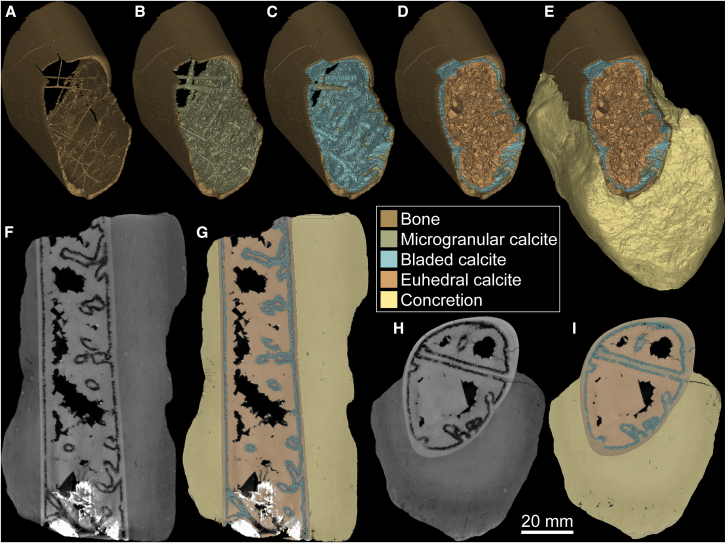
Three-dimensional models of partial anhanguerid pterosaur wing phalanx—MPSC R 1395—and its associated concretion and internal mineralization (A) Pterosaur wing phalanx, revealing numerous internal struts (most abundant on the lower margin of the element as it appears on the page, herein interpreted as the leading edge of the wing). (B) Pterosaur bone with proximal calcite layer depicted, revealing that it follows the internal surface of the bone (including each strut). (C) Pterosaur bone with proximal calcite layer and low-density layer depicted, demonstrating that the latter layer follows the topography of the internal surface of the bone and all but one of the proximal calcite-encased internal struts. (D) Pterosaur bone with proximal calcite layer, low-density layer, and crystalline calcite layers depicted, demonstrating the euhedral nature of the calcite crystals of the latter; the crystals did not fully occupy the medullary cavity, leaving some gaps that are best seen in (F–I). (E) Pterosaur bone with all layers depicted as in (D) but with the host concretion of the bone depicted as well. (F–I) μCT scans of the wing phalanx revealing (F and H) the density differences between the layers and (G and I) the density-based segmentation undertaken herein. (F) and (G) depict the section taken parallel to the long axis of the element; (H) and (I) depict the section taken perpendicular to the long axis of the element, revealing one of the few struts that spanned the diameter of the medullary cavity. All images are on the same scale; scale bars, 20 mm. Three-dimensional models have been derived from μCT data (Video S1).

Most elements of MPSC R 1395 were prepared mechanically and chemically.^35^^,^^36^ However, the phalanx studied herein was not acid-prepared prior to our analyses, and part of its host concretion remains present. μCT data reveal that the concretion is laminated and that numerous fossilized ostracods and a fish rib (evidently associated with soft tissue) are preserved as well (Figure 1 and Video S1). The density of the bone as per the μCT data is consistently higher than that of the surrounding or infilling matrix, enabling its relatively easy digital segmentation. Within the medullary cavity of the bone, three distinct and persistent mineralogical layers were segmented on the basis of gross morphology and apparent density: (1) a layer less dense than the bone, immediately proximal to it; (2) a low density layer; and (3) a dense, euhedrally crystalline layer (similar in density to the layer proximal to the bone).

Each of these layers was digitally segmented from the others based on density (Figure 1). Semi-quantitative X-ray diffraction (XRD) confirms the bone is primarily composed of fluorapatite (76.2%) and calcite (23.8%) (Table S1). The concretion is mainly calcite (88.0%) with amorphous silica (12.0%). Acid leachates of the fluorapatite contained incorporated sulfur and were also enriched in sodium, lithium, arsenic, chromium, strontium, zinc, copper, and molybdenum but depleted in manganese (Table S2) when compared with calcite leachates, indicating divalent dissolved manganese in the fluids.

### Early diagenetic transformation and bone soft tissue preservation

Partial microbial degradation of the fossilized bone was revealed through hydrogen and oxygen indices (HI and OI, respectively) of macromolecular organic matter measured by programmed pyrolysis (Table S1). The bone exhibited significantly lower HI values (9–38 mg hydrocarbon/g total organic carbon [HC/g TOC]) and higher OI values (89–298 mg CO_2_/g TOC) compared to the surrounding matrices and the host sediment from the Romualdo Formation (HI up to 810 mg HC/g TOC and OI ∼40 mg CO_2_/g TOC), indicating early degradation of organic matter of the carcass.^40^ The HI value of macromolecular organic matter typically decreases with its degradation,^41^ with the release of free H^+^ ions and organic acids (formic, acetic, and propionic acids) contributing to a localized increase in acidity.

The concretion-rich horizons of the Romualdo Formation are characterized by organic matter with high HI values (810 mg HC/g TOC),^33^ indicative of autochthonous deposition. Biomarker analyses demonstrate that it was deposited under euxinic, H_2_S-rich conditions.^26^ The anoxic environment has been implicated in preserving soft tissues by limiting microbial activity. However, the elevated OI in the pterosaur bone relative to the surrounding matrix suggests more oxidative conditions associated with the bone microenvironment. The organic matter composition and OI of the bone reflect a greater degree of diagenetic alteration than in the dispersed organic matter of the matrix, consistent with oxidative processes.

Exceptional soft-tissue fossilization, particularly via phosphatization, depends on a complex interplay of biological, microbial, and geochemical factors. For example, Wilby et al.^42^ demonstrated that the phosphatized mineralization of fossils in Jurassic limestones of Cerin, France, was facilitated by the phosphorus concentrated by microbial mats. Experimental work^18^ showed that intense microbial activity, particularly by sulfate-reducing bacteria (SRB), can lower pH during carcass decay, enabling phosphate precipitation. In contrast, soft-tissue preservation did not occur when microbial activity was limited.^18^ Certain taxa, however, appear less prone to phosphatization.

Other major influences on phosphatization include a supportive depositional environment, appropriately functioning microbiome, the amenable biochemical nature of degrading tissues, and a ready source of phosphate. Redox gradients identified across fossil remains and attributed to variations in the molecular composition and diagenetic transformation of different body parts have been implicated in the selective preservation of specific tissues of source organisms.^14^^,^^17^ The carotenoid-based coloration of a fossilized Miocene snake due to phophatization of pigment cells (xanthophores, iridophores, and melanophores) is a further example of the role of tissue type and chemistry in selective preservation.^43^ The mineralizing phosphate could be sourced autochthonously from the fossilized organism itself, e.g., from bones,^44^^,^^45^^,^^46^ or exogeneously, such as from microbial sources.^40^

Together, these studies show that while environmental conditions set the stage for exceptional preservation, biological factors—particularly tissue composition and its relative recalcitrance to microbial degradation—are decisive drivers of selective bone soft-tissue phosphatization.

### Mineralization, sulfur-oxidizing bacteria, and diagenetic transformation of organic matter

Microbial metabolisms in euxinic water conditions, as relates to the deposition and preservation of Romualdo Formation fossils, rely predominantly on sulfur cycling, which may have been stimulated by redox changes arising from initial degradation and diagenesis of the pterosaur. Sulfide is the predominant substrate for the metabolism of sulfur-oxidizing bacteria (SOB); both chemotrophic and phototrophic SOB (Equation 1) can convert reduced sulfur species into sulfate ions (SO_4_^2−^) and often coexist in suboxic and anoxic environments.^47^^,^^48^ These microbes are capable of growing under oxic and anoxic conditions, either with sulfur compounds or organic matter as an electron donor.^48^^,^^49^^,^^50^ Alternatives for oxygen as terminal electron acceptors for SOB include nitrate, Mn and Fe oxides, sulfide, quinone oxidoreductase, and NAD^+^.^51^^,^^52^^,^^53^ Chemoorganotrophic sulfide-oxidizing metabolisms are either coupled to degradation or the production of organic carbon, the former of which would result in a decrease in HI and increase in OI indices—as presently measured in the fossilized pterosaur bone.

**Figure 2:**
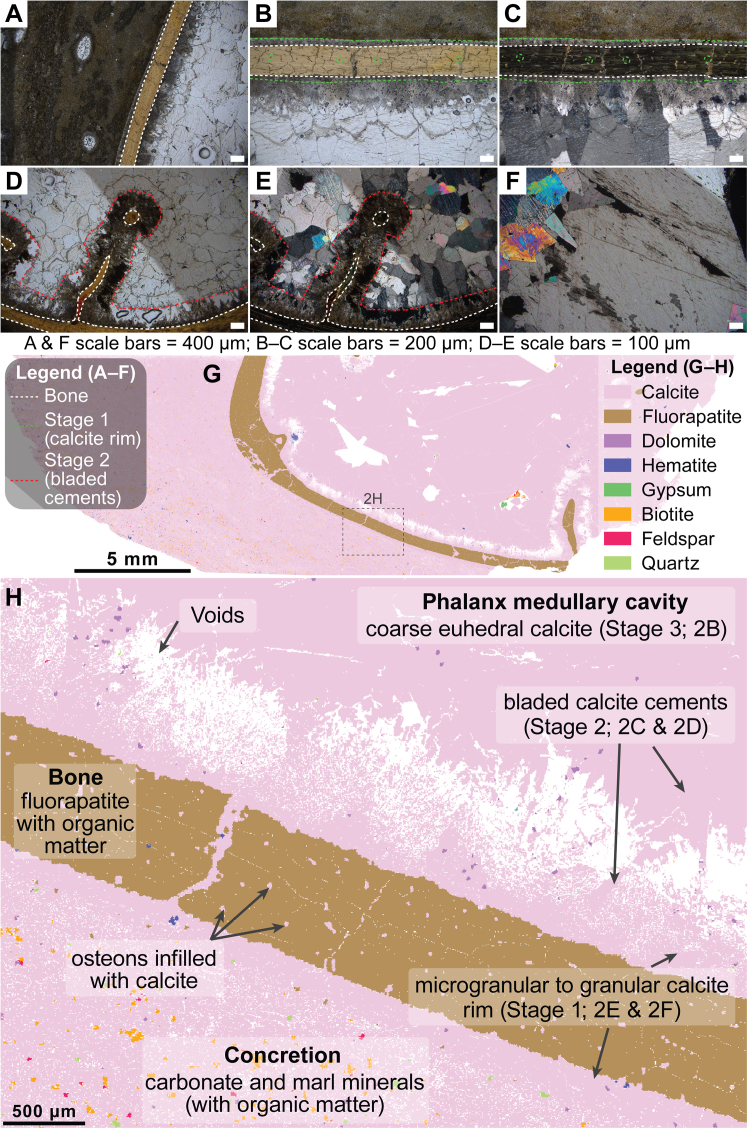
A progressive petrographic description of diagenetic infill stages in phosphatic pterosaur bone, moving through 3 stages (A) Primary phosphatic bone (tan). Lamellar structure + pores. Thin granular calcite cement rim externally. Thicker inner rim of granular → bladed cement. Plane polarized light (PPL), 2.5×. (B and C) Stage 1 (infill): green. Internal/external rims of turbid microgranular calcite. Possible replacement of precursor bladed-blocky phase. PPL and cross polarized light (XPL), 5×. (D and E) Primary fluorapatite trabeculae (tan). Rims: turbid microgranular → fine turbid bladed → inclusion-free coarse bladed → blocky. Relic/ghost structures from precursor bladed-blocky cement. Stage 2 (bladed cements): initial rims are ∼360–600 μm thick along trabeculae. Crystals are 150–200 μm, turbid/inclusion-rich. Transition to coarse inclusion-free bladed calcite (300–450 μm, up to 150 μm wide). Interdigitate → coarse blocky calcite. PPL and XPL, 2.5×. (F) Coarse crystalline calcite. Finer euhedral crystals (top-left). Stage 3 (final coarse euhedral crystals): infill center of cavity. Crystal sizes are 0.6–2 mm, up to 10 mm. Euhedral, blocky/equant. Clear, inclusion-free. Sharp or abrupt transitions in some zones (bladed ↔ 10 mm crystals). XPL, 2.5×. (G and H) Automated mineralogical mapping: fluorapatite represents bone; white represents pores/vugs. Three stages are: stage 1 (infill, granular/microgranular calcite), stage 2 (bladed calcite cements, transitional and aggradational growth), and stage 3 (final coarse euhedral crystals/blocky calcite infill) Automated mineralogical mapping for mineral-phase visualization. Stage 1 represents microgranular calcite in osteons, stage 2 represents bladed calcite (two types), and stage 3 represents coarse euhedral calcite in cavity. Matrix comprises carbonate + marl, porous + silicate-rich. The Tescan Integrated Mineral Analyzer (TIMA) does not resolve organic matter.

In syntrophic relationships between SOB and SRB, sulfur cycling is rapid and shifts to more reduced species, leaving a portion of sulfate unused.^54^ This excess sulfate accumulates in low or no oxygen niches and may form precipitates with several cations. Barite (BaSO_4_) and celestite (SrSO_4_) were identified directly inside and on the edges of the pterosaur bone (Figures 2 and S6–S10), whereas sulfate minerals were not detected in the surrounding matrix samples; barite together with gypsum was, however, identified within the euhedral calcite observed within the medullary cavity (Figures 2, S7, and S8). The association of barite and celestite with the bone, along with stable isotopic evidence (see the following sections), suggests the metabolic interplay between SOB and SRB. The sulfur isotope composition of total sulfur was measured in the bone and inner crystal samples (Table S1). When compared to contemporaneous seawater sulfate,^55^^,^^56^ the sulfur in the fossil bone (5.2‰ Vienna Cañon Diablo troilite [VCDT]) was depleted in the heavier ^34^S isotope indicating the contribution from the oxidation of formerly reduced sulfur species.

Celestite is a transition mineral between the carbonate and gypsum stages of evaporation in restricted marine environments, but it may also form via carbonate transformation.^57^ Upon deposition, this celestite can dissolve and disperse into marine sediments. Celestite can also be biogenically produced through the activity of cyanobacteria, sulfur bacteria in microbial mats, or SRB.,^58^ all of which are common in saline environments (e.g., seawater and hypersaline ponds). Celestite has previously been reported in the bone structure of fossils, notably from the Molí del Baró-1 site,^59^ where it was found in ten out of 13 specimens studied, with concentrations ranging from 4 to 89 weight (wt.) %. Strontium occurs naturally in bones and is a key element in paleontological studies, helping to understand diets, migration patterns, and the plants and animals that absorb strontium from the environment.^60^ It is likely that sulfate (SO_4_^2−^) from the activity of SOB and strontium ions available in the bone contributed to the high relative abundance of celestite conspicuous on the edges of the bone.

The δ^13^C signatures for the total carbonates in the pterosaur phalanx and matrix samples ranged from -10.4‰ to -13.1‰ (Figure S15 and Table S1). These ^13^C-depleted carbonates reflect a contribution to the bicarbonate pool from organic matter degradation by sulfur cycling microbes during early diagenesis,^61^ which reacted with Ca^2+^ to form calcite or contributed to the carbonate-content in apatite.^61^ Consistent with these data are the ^13^C-enriched isotope signatures for total organic carbon in the bone (-23.3‰), indicative of a loss of ^13^C-depleted biolipids from the bone upon fossilization. The δ^13^C of carbonates almost certainly includes contributions from calcareous carapaces of ostracods, which occur in high abundances within the surrounding concretion (Figure 1 and Video S1).

**Figure 3:**
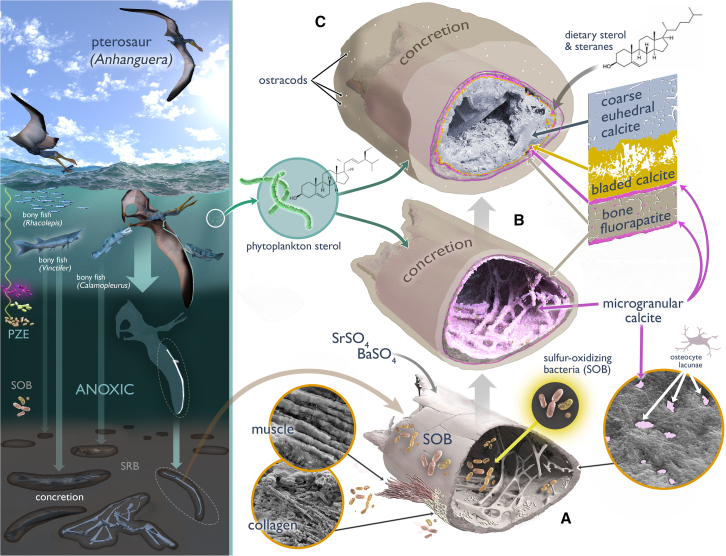
Reconstructions of the life and death of a pterosaur and the depositional environment of the Early Cretaceous rift basin of the Romualdo Formation Left: the life and death of a pterosaur and the depositional environment of the Early Cretaceous rift basin of the Romualdo Formation. The pterosaur was a predator of fish in surface water. Photic Zone Euxinia (PZE) refers to an anoxic-photic zone of the water column which is rich in H_2_S—produced by sulfate reducing bacteria (SRB) during anaerobic degradation of organic matter. Anoxygenic photosynthetic sulfur bacteria at the chemocline can utilize H_2_S as an electron donor in photosynthesis.^63^^,^^64^ Right: microbial and mineral fossilization pathway proposed for the 3D preservation of the pterosaur wing phalanx discovered in a concretion from the Romualdo Formation (Brazil). Separate stages comprise (A) initial degradation of the pterosaur body releases biochemicals (e.g., organic compounds with oxygen, low molecular weight (MW) fatty acids, or free H^+^) in the local environment, which stimulates certain microbial and mineralization processes^34^^,^^65^^,^^66^^,^^67^^,^^68^; (B) SOB oxidation of H_2_S produces SO_4_^2−^ which is thought to have reacted with metal cations (i.e., Ba^2+^and Sr^2+^) to produce mineral sulfates such as BaSO_4_ and SrSO_4_ identified close to the bone. Coincident phosphatization of the bone (and collagen) from hydroxyapatite to fluorapatite (also intimate with the fossilized bone) was likely facilitated by an increase in acidity. Carbonate precipitation enabled by subsequent redox fluctuations led to infilling of osteocytes by fine microgranular calcite; and (C) two further carbonate precipitation events contributed to bladed calcite and euhedral calcite layers of the concretion in which fossilized bone and organic matter were subsequently preserved.

After phosphatization, the bone material and ostracods from the water column^62^ experienced a three-stage cementation process (Figures 2 and 3), based on petrographic observations of the bone and associated cements. An initial stage of highly turbid microgranular to granular cement (<10–25 μm) lines the inner and outer phosphatic bone surfaces with rim thicknesses up to 100 μm, contemporaneous with calcite infill of post-depositional fractures (Figures 2A–2C). The second stage of calcite cementation is characterized by relatively fine (150–200 μm), turbid, bladed cements up to 360–600 μm thick that progresses to coarser (300–450 μm) inclusion-free bladed cement (Figures 2B–2E). Finer bladed cement is better developed around trabeculae/struts (Figures 2D and 2E). At the final stage (Figure 2F), coarse calcite (0.6–2 mm) with inclusion-free blocky-euhedral habits infill much of the medullary cavity. The very center of the bone cavity is filled with a few calcite crystals, up to 10 mm in size; however, this infilling was incomplete, leaving some voids in the cavity of the phalanx.^69^

Collectively, the isotopic signatures of organic matter (δ^13^C), sulfide minerals (δ^34^S) and biogenic carbonate (δ^13^C) minerals of varying grain sizes point to complex microbial implicated mechanism of mineralization and fossil preservation. Initial degradation and diagenesis of the source organism triggered the formation of mineral cements, which probably physically halted further molecular transformation, thus supporting the retention of biochemical material within the fossilized bone.

Episodic and localized oxidative conditions related to initial organic degradation of the pterosaur body provided biochemical nourishment of opportunistic microbes, including SRB accompanied and aided by SOB activity (inferred by barite and celestite precipitation). Related redox fluctuations drove phosphatized permineralization, and subsequent carbonate precipitations resulted in the comprehensive entombment of the fossil (concretion formation).

### Biomarkers and pterosaur diet

Molecular biomarkers efficiently sequestered within the concretion fossil before significant diagenetic transformation was evident from eukaryotic-sourced steranes detected in the lipid fraction extracted from all pterosaur and matrix samples. Biomarkers can provide valuable ecological and evolutionary information about ancient organisms. The samples showed a complex mix of steroids, with varying percentages of C_27_, C_28_, and C_29_ members, reflecting both marine and terrestrial (e.g., plants and fungi) food web sources.^70^^,^^71^

A distinctive series of diasteranes (rearranged steranes) were detected and observed to be considerably more abundant in the pterosaur bone than in the carbonate and shale samples (Table S1). The rearrangement of regular steranes into diasteranes is likely a consequence of acidic conditions prevailing during bone fossilization, similar to the acid-controlled rearrangement of steranes identified in other fossilized soft tissues e.g., sauropod dinosaur^72^ and fossil fish.^14^

Clays can also catalyze this steroid rearrangement,^73^^,^^74^ but no clays were identified in the samples by any of the petrographic or mineralogical analyses conducted. Recent concretion fossil research linked steroids (e.g., cholestane) intimately to iron carbonates, and not co-occurring phosphates,^75^ but the preferential association of steroids of the present pterosaur fossil to the microgranular to granular calcite cement (10–25 μm) of the host concretion needs to be confirmed.

Stable carbon isotopic values (δ^13^C) could be measured for cholesterol from the pterosaur bone (-19‰ VPDB, Table S3) and ethylcholesterol (C_29_ sterol) in the matrix (-29‰ VPDB, Table S3). The relatively ^13^C-enriched cholesterol of the pterosaur is consistent with the higher trophic level of the reptile and a fish-or cephalopod-based diet,^76^^,^^77^ while the more ^13^C-depleted ethylcholesterol in the matrix is attributed to phytoplankton in the paleo-water column.^76^

Hydropyrolysis (HyPy) release of covalently-bound hydrocarbons within the recalcitrant kerogen fraction,^78^^,^^79^ which has a very low vulnerability to hydrocarbons migrating from exogenous sources of the pterosaur bone produced mainly low molecular weight (C_11_–C_20_) normal and branched alkanes, but also low abundances of steranes (Figures S5A, S16, and S17). The sterane distribution was dominated by C_27_ cholestanes (87%), with traces of C_28_ (5%) and C_29_ (7%) steranes reflecting limited terrestrial influence.^70^^,^^71^ No steranes were detected in the pyrolyzed kerogen fraction of the distal matrix, indicating negligible eukaryotic input to the broader carbonate setting (Figure S5A).

**Figure 4:**
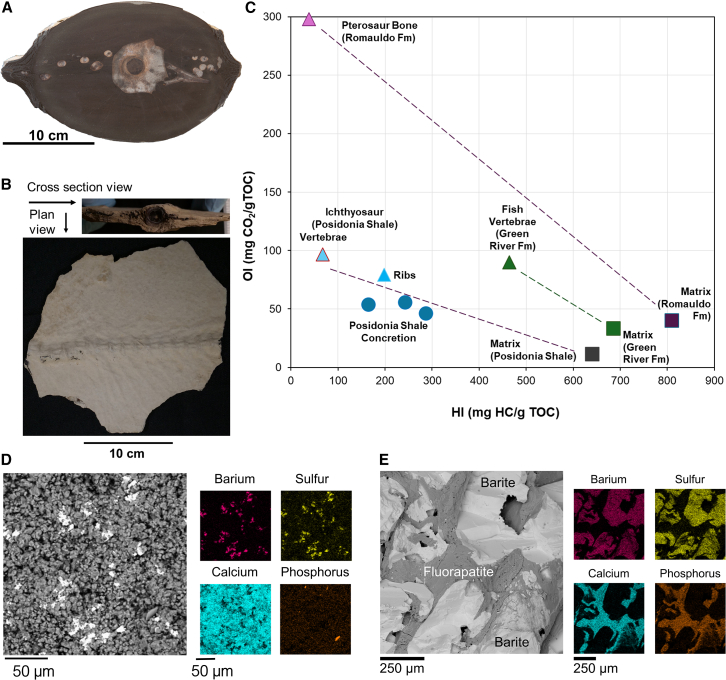
Rock-eval and mineralogical data from Posidonia Shale concretion with ichthyosaur vertebrae and ribs preserved and Eocene fish fossil with intact vertebrae and associated matrix, from the Green River Formation (A) Posidonia Shale concretion with ichthyosaur vertebrae and ribs preserved. (B) Eocene fish fossil with intact vertebrae and associated matrix, from the Green River Formation. (C) HI vs. OI across fossil and matrix components of three geological formations including the Romualdo Formation. (D) Barite and fluorapatite in close association shown via SEM analysis of HCl-treated fossil fish from the Eocene Green River Formation. (E) Barite observed in SEM analysis of HCl-treated fossil ichthyosaur vertebra from the Toarcian Posidonia Shale Formation.

Initial microbial degradation of the pterosaur biomass, such as attributed to stimulated sulfur cycling bacteria, must have been paused or shut-off at an early stage of steroid biomarker transformation. Other studies from the Eocene Green River Formation have shown that intact skin tissue of the fossil fish *Diplomystus dentatus* was preserved through phosphate permineralization in an oxygen-enriched microenvironment. OI revealed higher oxidation states in the skin than in vertebrae and bones, likely due to early degradation of the fatty acid-rich dermis. Barite was also found associated with the skin.^14^ Another *Diplomystus dentatus* fish and the Lower Jurassic Posidonia Shale (*Stenopterygius* sp.) (Figures 4 and S25; Table S4) have also similarly showed elevated acidity and oxidative indices adjacent to the preserved fossils and an intimate association with sulfate minerals. Evidence for organic fossil preservation via mineralization invoked by SRB and SOB activity enhanced by the redox gradients of initially degrading source organisms, has thus been found across different localities and geological periods. Definition here of this broadly occurring multi-mineral pathway of fossilization will help to unearth other exceptionally fossilized ancient organisms. More successful discovery of excellently preserved specimens will accelerate the biological and ecological information obtainable from this fossil resource and provide further opportunity to study the substrate and depositional dynamics most supportive of mineral preservation.

### Phosphatized nodules and collagen degradation

Scanning electron microscopy (SEM) of the carbonate demineralized (i.e., HCl treated) pterosaur bone revealed collagen fibers composed of fluorapatite and fluorite (Figures 2, S5C, S21, S22, and S23),^80^^,^^81^ with osteocytes previously infilled with fine microgranular calcite (Figures 2 and 3). The collagen displays a distinct crisscross pattern (Figures 3 and S21, 100–1,000 μm) typical of the collagen observed in modern birds^82^; this likely contributed to the pterosaur’s resilience to wind forces during flight.^83^

The fluorapatite and fluorite were arranged in an ordered array on collagen fibers (Figures S5E and S23), with focused ion beam (FIB) SEM and transmission electron microscopy (TEM) revealing them to be anuclear (Figures S5D, S5E, and S24). This ordered structure contrasts with the disordered anuclear nanocrystals observed in other fossils^84^ and other nodules composed of iron hydroxides.^85^ Such ordered collagen structure has been observed in modern birds where the collagen fibers form lamellae and occur among hydroxyapatite nano-sized crystals.^86^

Electron microscope–energy dispersive X-ray spectroscopy (EM–EDS) near the matrix surface also detected iron oxides with a mottled texture (Figures 3 and S22). The iron, possibly oxidized during acid treatment, may have originated from hemoglobin associated with muscle tissue attached to the bone.

SEM also revealed phosphatized microcrystals (composed of nanocrystals) in the bone after all acid treatments (results not shown). We propose that microbial involvement in the phosphatization process, likely including SOB and SRB, contributed to this mineralization. SOB thrive in sulfur and phosphate-rich environments, such as microbial mats^87^ and other marine surface sediments,^84^^,^^85^ helping to regulate acidic conditions that induce phosphate precipitation and mineralization.^88^^,^^89^ The formation of phosphatized nodules with microbial biofilms can encapsulate organic materials, contributing to fossil preservation. Bacteria mediate the formation of phosphate minerals like fluorapatite, catalyzing the authigenesis of minerals in the surrounding medium, often starting at the surface of bacterial cells or more commonly within the extracellular polymeric substance (EPS) matrix.^5^^,^^90^ EPS can be produced by a diverse range of bacterial microbes, and its extensive cation binding capacity is instrumental in the precipitation of phosphates^91^ as well as several other minerals.^87^^,^^92^

In the case of the pterosaur bone, microbial activity may have accelerated the formation of ordered fluorapatite and fluorite crystals,^93^ enhancing the preservation of organic remains such as collagen. The organic carbon of the collagen in the bone appears to have been transformed, as observed with other fossilized biomolecules.^84^ This process and the role of microbial activity in fossilization reflect the dynamic interaction between biotic and abiotic factors in the long-term geological preservation of biochemical material. Time-of-flight secondary ion mass spectrometry (ToF-SIMS) analyses of the HCl-treated bone showed no evidence of diagnostic collagen fragment ions identified from separate analysis of a collagen standard (Figures S13 and S14), and similarly, no collagen signal was detected from proteomic analyses, suggesting the original collagen was modified by processes such as phosphorylation, oxidation, and crosslinking during fossilization. A relatively high abundance of alkyl-aromatics were detected by HyPy analysis of the kerogen fraction of the bone (Figure S18), and these products may derive from the molecular transformation of collagen and lipid precursors during fossilization.^40^

### Limitations of the study

Limited sample material reduces the range of analyses that can be conducted on such a specimen. Further, destructive analysis limits the amount of material available for analyses.

## Resource availability

### Lead contact

Requests for further information and resources should be directed to, and will be fulfilled by the lead contact, Kliti Grice (k.grice@curtin.edu.au).

### Materials availability

No new materials were generated in this study.

### Data and code availability

• All data reported in this paper will be shared by the lead contact upon request.
• This paper does not report original code.
• We do not have any relevant key resources for table.

## Acknowledgments

The authors thank Curtin University for strategic funding for an Agilent 8890 gas chromatograph coupled to an Agilent 7010C triple quadrupole mass selective detector. This work was funded by the Australian Research Council Laureate Fellowship (FL210100103 to K.G.); the Australian Research Council Infrastructure (LE190100053 to K.G. and W.D.A.R., LP0991658 to K.G. and P.F.G., LE110100119 to K.G., and LE0882836 to K.G. and P.F.G.); the German Research Foundation (Schw554/29-1 to L.S. and Bo1548/8 to M.E.B.); the Conselho Nacional de Desenvolvimento Científico e Tecnológico (CNPq #406779/2021-0, CNPq #406902/2022-4, and CNPq #308707/2023-0 to A.W.A.K. and CNPq #309245/2023-0 to J.M.S.); and the Fundação Carlos Chagas Filho de Amparo à Pesquisa do Estado do Rio de Janeiro (FAPERJ; #E-26/201.095/2022 to A.W.A.K., and #E-26/210.066/2023 and #E-26/204.280/2024 to J.M.S.).

## Author contributions

Conceptualization, K.G.; field work and collection of the specimens, K.G., S.F.P., L.S., A.L.E., A.J.Y.J., A.A.F.S., R.A.M.B., A.H.P., J.M.S., and A.W.A.K.; methodology, K.G., S.F.P., L.S., M.A.D.M., P.F.G., L.M.B., M.T., A.L.E., A.J.Y.J., A.I.H., M.E.B., P.H., X.S., A.D., A.V.d.O., W.D.A.R., H.R.-B., and I.S.; investigation, K.G., S.F.P., L.S., A.A.F.S., R.A.M.B., and A.W.A.K.; visualization, K.G., S.F.P., and V.O.L.; funding acquisition, K.G.; project administration, K.G., L.S., and A.W.A.K.; supervision, K.G.; writing – original draft, K.G., S.F.P., L.S., P.F.G., J.D., A.H.P., M.E.B., P.T.V., and R.H.C.M.; writing – review and editing, K.G., S.F.P., L.S., M.A.D.M., P.F.G., L.M.B., M.T., A.L.E., A.J.Y.J., A.A.F.S., R.A.M.B., J.D., A.I.H., M.E.B., A.H.P., R.H.C.M., P.H., X.S., A.D., A.V.d.O., P.T.V., L.S., and A.W.A.K.

## Declaration of interests

Authors declare that they have no competing interests.

## STAR★Methods

### Method details

Data S1/Methods S1 provides details on the analytical methods employed on the samples.

#### Sample and sampling location

Figure S1 shows location of sample collection (red dot) is Sítio Baixa Grande.

#### MicroCT scanning

The specimen was scanned using a Zeiss Versa XRM 520 3D X-ray microscope equipped with a flat-panel detector and installed at the Australian Resources Research Center (CSIRO Mineral Resources, Kensington, Western- Australia). The instrument was set up to maximize phase contrast between the various constituents of the specimen (160 kV, 10 W and LE3 filter). A total of 2001 projections (10 frames per projections) were recorded over a 360° rotation of the specimens to reconstruct its 3D volume with a voxel size of 21.9 μm (Video S1).

#### Samples

Four samples (Figure S2) were obtained for carbonate δ^13^C and δ^18^O, δ^13^organic matter rock eval pyrolysis (HAWK) and biomarker analyses by hydropyrolysis (HyPy), gas chromatography – mass spectrometry (GC-MS), GC-metastable reaction monitoring (MRM) and compound specific isotope analyses (CSIA) (Figures S2 and S3).

Additional samples from the Green River Formation and Posidonia Shale were obtained to compare with the pterosaur samples across other geological time intervals and environments. These samples underwent rock eval pyrolysis (HAWK) (Table S4). A delicately preserved fish fossil with multiple vertebrae, and associated sedimentary matrix, was collected from the 18-inch layer of the Green River Formation—an Eocene lacustrine lagerstätte.^94^

The sample was carefully separated using a tungsten carbide scribe (with tips sonicated in dichloromethane [DCM]:methanol [MeOH], 9:1 v/v, 15 min × 3) and ground to a fine powder using a pestle and mortar, then extracted in a microwave as described below along with activated copper turnings (4 M HCl) to remove elemental sulfur. Programmed pyrolysis was performed on the extracted residues (see below).

A partial specimen of *a Stenopterygius* sp. (*Ichthyosauria*) consisting of vertebrae and ribs encased within a calcitic concretion was excavated from a Posidonia Shale quarry in Dormettingen (Baden-Württemberg, Germany). The Posidonia Shale is a ∼183–180 Ma (Toarcian) marine Konservat-Lagerstätte.^65^The concretion was sampled by drilling 25 mm-wide core plugs into the dorsal, middle and ventral portions, then cutting the vertebrae and rib pieces from the matrix. The fossil bones, concretion and shale samples were ground to fine powder with a pestle and mortar or a ring mill pulverizer. All samples were microwave extracted as described below and desulfurized with activated copper turnings. Programmed pyrolysis was performed on the extracted residues (see below).

#### Preparation of samples

The hard cortical bone (sample 1, as shown in Figure S2), was first separated from the surrounding carbonate matrix. Small pieces of the cortical bone were cut using a Dremel tool fitted with a pre-cleaned diamond blade. The diamond blade was sonicated three times (each for 15 min) in a 9:1 vol/vol mixture of dichloromethane (DCM) and methanol (MeOH) to ensure it was free from contamination before use. The bone pieces were then rinsed thoroughly in DCM to remove any surficial contamination or external impurities. After rinsing, the bone pieces were manually powdered using an annealed (500°C, 2 h) porcelain mortar and pestle.

Two matrix samples were similarly prepared: sample 3 was taken from near the pterosaur bone, and sample 4 was taken from the distal part of the matrix. Both of these matrix samples underwent the same preparation steps as the hard cortical bone above. Finally, a sample of calcite crystals (sample 2), which had partially filled the hollow region of the bone, was processed in a similar manner.

#### Samples for biomarker, pyrolysis, elemental and stable isotopic analyses

A summary of the analytical protocol for the four samples described above is presented in Figure S3.

### Quantification and statistical analyses

Not applicable.

## Supplemental information

Document S1. Figures S1–S25, Tables S1–S4, Data/Methods S1, Equation 1
