# Supplementary material for: Multi-staged mineralization and biomarker preservation in a 113-million-year-old pterosaur bone via redox shifts in diagenesis
Source: iScience. 2026 Jun 18;29(7):116199. doi: 10.1016/j.isci.2026.116199 (PMC13377889; doi:10.1016/j.isci.2026.116199)
Supplement: Document S1. Figures S1–S25, Tables S1–S4, Data/Methods S1, Equation 1 [file mmc1.pdf]

iScience, Volume *29*

Supplemental information

Multi-staged mineralization and biomarker preservation in a 113-million-year-old pterosaur bone via redox shifts in diagenesis

Kliti Grice, Stephen F. Poropat, Lorenz Schwark, Maria A. Diaz Mateus, Paul F. Greenwood, Luke M. Brosnan, Madison Tripp, Amy L. Elson, Andrew J.Y. Jian, Antônio A.F. Saraiva, Renan A.M. Bantim, Julien Demore, Alex I. Holman, Michael E. Böttcher, Adele H. Pentland, Robert H.C. Madden, Peter Hopper, Xiao Sun, Aaron Dodd, Arthur V. de Oliveira, Pieter T. Visscher, William D.A. Rickard, Juliana M. Sayão, Hossein Rahimpour-Bonab, Iris Schmiedinger, Victor O. Leshyk, and Alexander W.A. Kellner

Other Supplementary Materials for this manuscript include the following:

Data/Methods S1 section

Samples for biomarker, pyrolysis, elemental and stable isotopic analyses

A summary of the analytical protocol for the four samples described above is presented in fig. S3 and outlined briefly below.

Extraction and fractionation

Powdered samples (ca. 5–10g) were extracted with a mixture of 9:1 vol/vol DCM and MeOH (9:1 vol/vol, 30 mL) using a Milestone Ethos X Advanced microwave extraction unit using two cycles of heating to 110 ºC over 15 min, 10 min static time, and 30 min cooling time, changing the solvent between cycles. The extracts were combined, evaporated under N_2 at 35 °C, left overnight with activated Cu turnings (4 M HCl) to remove elemental sulfur, and filtered through anhydrous $MgSO_4$ to remove residual water and fine particles. The filtered extracts were evaporated to dryness and weighed.

The extracts were then fractionated via liquid chromatography on activated silica gel (160 °C overnight, 5.5 cm, 0.5 cm i.d.). Saturated hydrocarbons were eluted with *n*-hexane (4 mL), aromatic hydrocarbons with *n*-hexane and DCM (7:3 vol/vol, 4 mL), and polar compounds with DCM and MeOH (1:1 vol/vol, 4 mL). The saturated and aromatic fractions were evaporated to dryness and dissolved in *n*-hexane to 1 mg/mL for analysis.

Polar fractions were derivatized using bis(trimethylsilyl)-trifluoroacetamide (BSTFA) and anhydrous pyridine (for 100 µg, 60 µL BSTFA, 40 µL pyridine) heated at 60 °C for 30 minutes, and dried under a nitrogen purge. The derivatized fractions were dissolved in *n*-hexane and analyzed using GC-MS and CSIA.

Hydropyrolysis (HyPy) of extracted residues

Extracted rock powder (ca. 0.8 g of hard bone sample 1 and 0.9 g of distal matrix sample 4) from each sample 1, 3 and 4 (fig. S2) was mixed with ammonium dioxydithiomolybdate catalyst $(NH_4)_2MoO_2S_2$, 5 % dry mass) and pyrolyzed using a commercial HyPy apparatus (STRATA Technology LTD) following standard procedures [1,2]. Briefly, the sample was heated from 25 °C to 250 °C at 300 °C/min, then from 250 °C to 520 °C at 8 °C / min and held for 2 minutes. A constant pressure (15,000 kPa) and flow (5 L/min) of ultra-high purity hydrogen (Coregas) was maintained throughout thermal treatment. The released compounds were cryogenically trapped on a silica-filled trap cooled with dry ice. Products adsorbed on the silica trap were eluted with DCM: MeOH (9:1 vol/vol, 20 mL) on a large chromatographic column, and elemental sulfur was removed with activated copper turnings (4M HCl). Between analyses, the reactor and trap were cleaned as follows: (i) 2 × 15 minutes of ultra-sonication in a 9:1 vol/vol DCM:MeOH mixture. (ii) Sample-free HyPy treatment: Heated from 25°C to 300°C at 300°C/min, then from 300°C to 550°C at 10°C/min, held for 5 minutes under constant pressure (15,000 kPa) and H_2 flow rate (5 L/min). The system was confirmed to be analyte-free before each analysis by pyrolyzing catalystloaded silica gel.

HyPy was employed to extract covalently bound steroids from both the pterosaur bone and the distal carbonate matrix. The bone primarily yielded C_{27} cholestane, supporting a predominantly fish/ cephalopod based diet for the pterosaur confirmed by CSIA. By contrast, the distal carbonate matrix showed an absence of steroid preservation, indicating minimal retention of organic matter. Additionally, the HyPy analysis revealed the presence of breakdown products like alkylbenzenes and alkylphenanthrenes, which may result from the degradation of collagen and/or carotenoids.

GC-MS and GC-MRM analyses

Saturated and aromatic fractions of all four bitumens and two HyPy products (samples 1 and 4) were analyzed by gas chromatography–mass spectrometry (GC–MS). Saturates were analyzed using an Agilent 8890 gas chromatograph (GC) coupled to an Agilent 5977C mass-selective detector (MSD), fitted with a DB-1 MS ultra-inert column (60 m, 0.25 mm i.d., 0.25 µm film

thickness). Aromatic fractions were analyzed using an Agilent 7890B GC coupled to an Agilent 5977B MSD, fitted with a DB-5 MS ultra-inert column (60 m, 0.25 mm i.d., 0.25 µm film thickness). All fractions used the same inlet conditions and oven program. One µL of sample was injected into a split-splitless injector operating in pulsed splitless mode (inlet temperature 280 °C, 1.1 mL / min He carrier gas flow). Oven temperature was held at 40 °C for 1 min, heated at 3 °C / min to 325 °C, then held for 30 min. Data was collected in full scan and selected ion monitoring (SIM) mode simultaneously, and processed with Agilent ChemStation software. Ions extracted included 128, 142, 156 for alkylnaphthalenes and 178, 192, 206 alkylphenanthrenes.

Sterane biomarkers were analyzed by metastable reaction monitoring (MRM) using an Agilent 8890 GC coupled to an Agilent 7010C triple quadrupole MSD, fitted with a DB-5 MS ultra-inert column (60 m, 0.25 mm i.d., 0.25 µm film thickness). 1 uL of sample was injected into a multimode injector operating in pulsed splitless mode (inlet temperature 280 °C, 1.5 mL / min He carrier gas flow). Oven temperature was held at 60 °C for 2 min, increased to 220 °C at 8 °C / min, then to 320 °C at 2 °C / min, and held for 28 min. The measured MRM transitions were $M^{\bullet+}$ → 217.2 for C_{27} to C_{29} steranes. MRM transitions monitored in aromatic fractions: isorenieratane (546.5 → 134.1); aryl isoprenoids (134.1 → 119.1). Data was processed using Agilent MassHunter software.

<u>Compound-specific isotope analysis (CSIA) of sterols</u>
CSIA ($\delta^{13}C$) of the derivatized polar fractions and CSIA of saturated and aromatic compounds after HyPy were performed using a Thermo Delta V Advantage isotope ratio mass spectrometer (irMS), coupled to a Thermo Trace GC Ultra via a GC Isolink interface. GC conditions were consistent with those used for the GC–MS analysis (described above). The outflow from the GC column passed through the GC Isolink combustion reactor (containing copper oxide and nickel oxide at 1000 °C), where the hydrocarbons were combusted to CO_2. The irMS measured the ions at *m/z* 44, 45 and 46. $\delta^{13}C$ was calculated by Thermo Isodat software and calibrated to the Vienna Pee Dee Belemnite carbon isotope scale, based on an in-house standard that included a mixture of n-alkanes (*n*-C_{11}, *n*-C_{13}, *n*-C_{14}, *n*- C_{17}, *n*-C_{18}, *n*-C_{19} and *n*-C_{25}; with known isotopic compositions ranging from -25.3 to -32.2 ‰. Several samples were selected based on signal strength and were analyzed for compound- specific carbon isotopes. Average values and standard deviations for each compound were calculated.

A pure cholesterol standard (underivatized and derivatized) was analyzed to calculate the $\delta^{13}C$ of the additional methyl-groups from BSTFA. Samples were run as triplicates and the $\delta^{13}C$ values of the parent compounds were corrected for the isotopic composition from the methyl-groups of the BSTFA.

The steranes present in the bone included C_{27}, C_{28}, and C_{29} compounds, which are indicative of various sources, including phytoplankton and terrestrial plants/fungi. The isotope data provided insights into trophic levels, with the cholesterol of the pterosaur reflecting a higher trophic level compared to the other sterols in the matrix.

<u>Rock eval pyrolysis</u>
Total organic carbon (TOC) analysis and programmed pyrolysis were performed on all four extracted samples. Programmed pyrolysis provides multiple parameters for OM characterization [3]. This encompasses: hydrogen indices (HI) (hydrocarbons generated from the kerogen, relative to total organic content (TOC) and /expressed as mgHC/g TOC), oxygen indices (OI) (quantity of CO_2 generated from the kerogen, relative to TOC and expressed as mgCO_2/g TOC), S1 (quantity of free hydrocarbons expressed as mgHC/g of rock), S2 (hydrocarbon generative potential expressed as mgHC/g of rock), Tmax (temperature of maximum pyrolysate yield expressed in °C), TOC amount of OM expressed in wt. % and carbonate carbon expressed in wt. %. Programmed pyrolysis was undertaken with a Wildcat Technologies Hydrocarbon Analyzer workstation (HAWK®) following standard protocol [3].

Hydrogen and Oxygen Indices (HI and OI) were measured through programmed pyrolysis via a WILDCAT HAWK workstation, which is commonly used for analyzing organic matter in sedimentary rocks. The pterosaur bone and its surrounding matrix samples showed distinct

hydrogen index (HI) and oxygen index (OI) values, indicating oxidative microbial degradation of organic biopolymers within the bone.

Acid treatment of bone samples

One piece of pterosaur bone was soaked in dilute HCl (5 M) for one week. The bone was carefully washed with ultrapure water (18 MΩ cm), methanol and dichloromethane and dried with a N_2 purge. In another experiment, two pieces of bone were placed in three different petri dishes containing various acids (20 mL) for 24 hours (fig. S4). Acids used were light acetic acid (7 %), dilute HCl (0.05 M) and acetate buffer (pH 3.8) [4]. The bones were carefully washed with ultrapure water, methanol and dichloromethane and dried with an N_2 purge. Samples were coated with platinum for SEM and FIB-SEM.

Bones from Green River Formation and Posidonia Shale were treated with dilute HCl (0.05 M). The bones were carefully washed with ultrapure water, methanol and dichloromethane and dried with an N_2 purge. Samples were coated with platinum for SEM and FIB-SEM.

Other Techniques

For SEM-EDX analysis, sample 1 was also treated with 0.5 M HCl, washed and dried and the residue was investigated via SEM-EDX [5]. The samples were analyzed at IOW with a scanning electron microscope (SEM Merlin-Compact MV, Zeiss) coupled to an energy-dispersive X-ray microanalysis system 316 (EDX, Oxford Instruments).

X-ray diffraction (XRD)

Ground residues of the bone sample 1 and sample 4 calcium carbonate matrix distal to the hard bone were subjected to semi-quantitative XRD analyses (table S1). Sample 1 was suspended in ethanol and deposited on a low-background mount, and sample 4 was mounted using the side packing method, Samples were analyzed using a Bruker-AXS (Germany) D8 Advance powder diffractometer with a Cu Kα radiation source (1.5046 Å) and a LynxEye position-sensitive detector. Data was collected over the range 5° to 90° 2θ with a step size of 0.02° and a collection time of 0.7 seconds per step. A 0.3° divergence was used with 2.5° Soller slits in the incident and diffracted beams. Crystalline phases were identified using the search / match algorithms implemented in DIFFRAC.EVA 5.2 (Bruker-AXS, Germany) to search the International Centre for Diffraction Data Powder Diffraction File (PDF5+ 2024 edition). The relative proportion of identified crystal phases was determined by Rietveld analysis conducted using the TOPAS v.7 software (Bruker-AXS, Germany).

Bulk $\delta^{13}C$ of organic matter

Aliquots of powdered and extracted sample were stirred with HCl (1 M) until bubbling ceased, then left overnight to remove all carbonates, washed with Milli-Q water, and freeze-dried. Decarbonated powder was weighed in triplicate into tin cups (Elemtex) and analyzed for bulk $\delta^{13}C$ using a Thermo Scientific Flash 2000 HT Elemental Analyzer (EA) coupled to a Thermo Delta V Advantage via a Conflo IV interface. Samples were combusted to CO_2 in the Flash 2000 HT combustion furnace (chromium oxide, reduced copper, silvered cobaltous-cobaltic oxide) at 1020 °C. The irMS measured the ions at *m/z* 44, 45 and 46. $\delta^{13}C$ was calculated by Thermo Isodat software and normalized to the Vienna Pee Dee Belemnite carbon isotope scale using the standard reference materials NBS-19 (+1.95 ‰) and L-SVEC (-46.6 ‰) [6]. Measurement accuracy was assessed using the standard reference material IAEA-600 (-27.77 ‰).

$\delta^{34}S$ of total sulfur (TS)

For measuring contents and the stable isotope composition of TS, dried and homogenized samples were weighed in pre-cleaned tin cups and combusted in a Thermo Scientific Flash EA Isolink Elemental Analyzer connected to a Thermo Finnigan MAT 253 gas mass spectrometer via a Thermo Conflo IV split interface [7]. The results are given in the conventional δ-notation, where the given results in [‰] are equivalent to [mUr] (milliUrey) [8]. Sample gas was transported in a stream of high grade (5.0) helium. International isotope intercomparison materials were used to

convert the isotope ratios to the VCDT scales following Mann et al. [9] with a precision of better than ± 0.3‰ ($\delta^{34}S$).

Inductively Coupled Plasma Optical Emission Spectrometry (ICP-OES)
For all four samples the acid-extractable fraction of Ca, Mg, Fe, Mn, Sr, Na, K and Al was measured (table S2) after the reaction of powdered dry sediment with 0.5 M p.a. grade HCl for 1 hour at room temperature [10]. The extracts were filtered through disposable cellulose acetate membrane filters (0.45 µm pore size), and analyzed by inductively coupled plasma optical emission spectrometry, ICP-OES (Thermo, iCAP 7400 Duo, Thermo Fischer Scientific), after appropriate dilution with HNO_3 using external calibration and Sc as an internal standard.

$\delta^{13}C$ and $\delta^{18}O$ of carbonate
$\delta^{13}C$ and $\delta^{18}O$ values of the carbonate fraction were measured on CO_2 liberated by the reaction with H_3PO_4 at 72°C with a precision of better than ±0.15‰ [11]. Reaction took place in a Thermo Gasbench II connected to a Thermo Finnigan MAT 253 gas mass spectrometer via a Thermo Conflo IV spilt interface. Scaling of measured carbon and oxygen isotope results to the VPDB scale took place via carbonate reference materials (NBS19 and LVSEC) [6].

Polished thin section preparation
The sample was initially sub-sampled to an appropriate thin section block using a precision diamond wheel at very low RPM allowing for no coolants or lubricants to be used whilst maintaining low risk of heating and chipping. A new diamond wheel was used for this preparation and the cutting surface of the saw blade was pre-conditioned using a pure quartz sand conditioning block and subsequent cleaning with deionized water was conducted to ensure no factory/production materials were embedded.

The final block was exceptionally fragile and brittle. A thin epoxy was applied to external friable surfaces to maintain sample integrity during subsequent trimming and polishing stages of thin section production. The face of each prepared block was polished to a ~1200 grit flat surface to produce a suitable thin section billet and mounted to a frosted glass microscope slide with RenLam epoxy resin and hardener. Thin section billets were cured to the glass slides in a spring activated mounting fixture for 24 hours. Using a vacuum jig, the glass mounted billets were trimmed of excess material using a diamond trim saw lubricated with deionized water. Billet trimming yields a billet thickness of ~500-1000 µm. The saw blade, and cutting table were cleaned thoroughly between samples to remove contaminants and debris. Automated lapping wheels with abrasive slurry feeds removed excess material from the trimmed billet to a thickness of slightly over 30 µm. Sample specimen holding fixtures and vacuum jigs were utilized to ensure the production of a ground finished thin section with even thickness. The final thinning of the thin section to 30 µm and production of a polished surface was completed in progressive stages: (1) 9 µm polish using Struers DP Suspension P9 mic with DP Green lubricant on an MD Plan cloth, (2) 3 µm polish using Struers DiaPro Dac 3-micron suspension and Kemet Liquid polish suspension 3-micron on a Kemet ASFL cloth, and (3) a 1 µm polish using Struers DiaPro Nap B suspension and Kemet Liquid Polish 1-micron suspension on Struers MD Nap, MD Floc and Kemet ASFL cloths.

Carbonate petrology
Descriptions of carbonate cements and their spatial relationships with the fossil bone material were made principally from the polished thin section examined using a Nikon Eclipse LV100N Pol petrographic microscope with plane and cross polarized transmitted (and reflected) light at magnifications of 25-500x. Photomicrographs were collected using MIchrome 20 digital camera and referenced against a thin section map for subsequent analyses. Descriptive terminology of carbonate phases broadly follows that of Flügel [12].

Tescan Integrated Mineral Analyzer (TIMA)
Polished thin section (fig. S2) was analyzed using the TESCAN Integrated Mineral Analyzer (TIMA). The TIMA system comprises the TESCAN Mira3 Field Emission-SEM coupled with four

energy dispersive (EDS) detectors. Polished thin section was carbon coated and scanned using liberation analysis (EDS spectroscopic data are collected along with the BSE signal simultaneously) via dot mapping (electron beam steps through an equally spaced grid over the area) using pixel spacing (BSE grid) of 3 μm and dot spacing (EDS grid) of 15 μm. Analyses were conducted at operation conditions of 25 kV accelerating voltage, 18.48 beam intensity, 5.69 nA probe current, 15 mm working distance, and 85 nm beam spot size. For phase identification, the spectroscopic data was matched to mineral definition files from the TIMA software. For refinement of phase identification, unclassified and mismatched spectra were matched to reference spectra from the mineral database in the TESCAN TIMA 2.9.0 software.

Time of Flight-Secondary Ion Mass Spectrometry (ToF-SIMS)

ToF-SIMS analyses were performed on an M6 instrument (ION-TOF GmbH, Germany) [13,14]. A high-resolution bismuth liquid metal ion gun (LMIG) was operated using a 30 keV Bi^+ ion source with a pulsed beam current of approximately 0.1 to 0.7 pA over areas varying from 400 × 400 μm^2 to 500 × 500 μm^2, with 512 × 512 to 1024 × 1024 pixels per frame. An electron flood gun with low energy electrons was used for charge compensation. Each analysis location was sputter cleaned *in-situ*, using an Ar gas cluster ion beam (GCIB) (2.5 keV, median 2000 atoms) (Figs. 2B(i), S11A,C) to remove surface organic contamination, or using the $O2^+$ source (2 keV, 1uA) (Figs. 2B(ii), S10, S11B,D) and Cs^+ source (2 keV, 1uA) (Fig. S11D) of the dual source column (DSC) to remove surface contaminants and enhance the positive and negative secondary ion yield, respectively.

Data was analyzed using SurfaceLab version 7.4. Mass spectra were calibrated using a combination of four or five of the following secondary ions in positive ion acquisition: Fe^+, CaO^+, Ca_2O^+, $CaPO_2^+$, $Ca_2O_2^+$, $Ca_2PO_4^+$, and $Ca_3O_3^+$, and four or five of the following secondary ions in negative ion acquisition: CaO^-, $CaOH^-$, PO_2^-, SO_2^-, PO_3^-, and SO_3^-. Maps have been adjusted for contrast by adjusting minimum and maximum intensities.

Collagen standard preparation and characterization

A pure standard of Collagen I powder (Collagen from bovine achilles tendon, Sigma Aldrich) was characterized using ToF-SIMS. Analyses were focused on positive secondary ion acquisition, as most characteristic protein fragments have been determined to be produced as positive ions [15,16].

Analyses were performed in high mass resolution (spectrometry) mode. The region of interest was pre-sputtered with an argon cluster source (cluster size of 2500 atoms, current of approximately 0.5 nA) at a raster size of 500 x 500 μm^2 for 10 second, to remove surficial hydrocarbon contamination. All high mass resolution analyses were performed below the static limit of 1 x 10^{13} ions/cm^2 to maximize secondary ion yield of molecular species [15,17]. The LMIG was operated using a 30 keV $Bi3^+$ ion source with a pulsed beam current of 0.2 pA over an area of 150 x 150 μm^2. An electron flood gun with low energy electrons was used for charge compensation.

Data was analyzed using SurfaceLab version 7.4. An advanced time of flight correction was applied to the acquired spectrum to correct for topographic effects caused by the crystal structure of the collagen powder. Mass calibration was performed using the following ions: NH_4^+, $C_2H_3^+$, C_2H_{5+}, $C_6H_4N_+$, C_5H_{7+}.

The spectrum for collagen was collected up to a mass of 1000 amu, however peaks only occurred up to approximately 600 amu (Fig. S12), suggesting that collagen fragments primarily into sub-units of this mass. Peaks in the low molecular weight region of the collagen spectrum were assigned based on comparison of exact atomic mass with expected masses of hydrocarbon and N-containing organic ions and selected according to known organic ions characteristic of proteins (e.g., proteins identified in 64) (fig. S13).

Collagen Alteration: The characterized ToF-SIMS and SEM-EDS analyses showed that collagen fibers in the bone, and ostracods in the matrix, were well-preserved as fluorapatite. However, their organic components (e.g., amino acids) were unable to be identified in the acid-treated bone and therefore were probably significantly altered by chemical processes during fossilization.

Elevated sulfur % were found in both the bone and ostracods compared with the surrounding matrices.

Scanning Electron Microscopy (SEM)
Microscopic observations and elemental composition analyses were conducted using scanning electron microscopy (SEM) and X-ray energy-dispersive spectroscopy (EDS) on a Tescan LYRA3 GM instrument to prevent charging and enhance the secondary electron signal, samples were coated with a 5 nm layer of platinum. SEM imaging was performed at acceleration voltages of 5 kV and 10 kV, while EDS analyses were conducted at 20 keV with a working distance of 20 mm for 100–120 seconds.

Focused Ion Beam Scanning Electron Microscope (FIB-SEM)
The acid treated bone samples were examined using a Tescan Lyra FIB-SEM. The bone was mounted onto an aluminum stub and coated with carbon. A cross-sectional lamella covering several of the phosphatized nodules (fig. S22) was extracted using standard FIB-SEM lift out techniques, mounted onto a copper grid and thinned to ~100 nm, followed by a low kV (2 kV) 'clean up' routine to remove surface damage.

Energy Dispersive X-ray Spectroscopy (EDS), and SEM-EDX revealed the presence of celestite ($SrSO_4$) and barite ($BaSO_4$) in the pterosaur bone, supporting the hypothesis of microbial sulfur oxidation. These minerals form when sulfate ions interact with barium or strontium ions in the surrounding water or sediment. Sr could also be derived from the pterosaur's diet.

Phosphatized nodules made of fluorapatite and calcium fluorite, likely formed by microbial activity, were observed around the collagen fibers (fig. S23, S24). Focused ion beam scanning electron microscopy (FIB-SEMS) revealed anuclear fluorapatite and calcium fluorite nodules after dilute HCl acid treatment, ranging in size from approximately 0.05 μm (nanocrystals) to 5 μm (consisting of nanoclusters), and covering the surface of the collagen fibers (fig. S5D).

Transmission electron microscopy (TEM) and scanning transmission electron microscopy (STEM)
Lamellae prepared by FIB milling were examined by transmission electron microscopy using a ThermoFisher Talos F200FX microscope equipped with Super-X EDS detectors. The microscope was operated at 200 kV. Low and high-resolution imaging was performed using TEM and STEM, which was coupled with EDS for elemental composition analysis. Diffraction patterns were taken for further crystallographic characterization using selected area electron diffraction (SAED) (fig. S25).

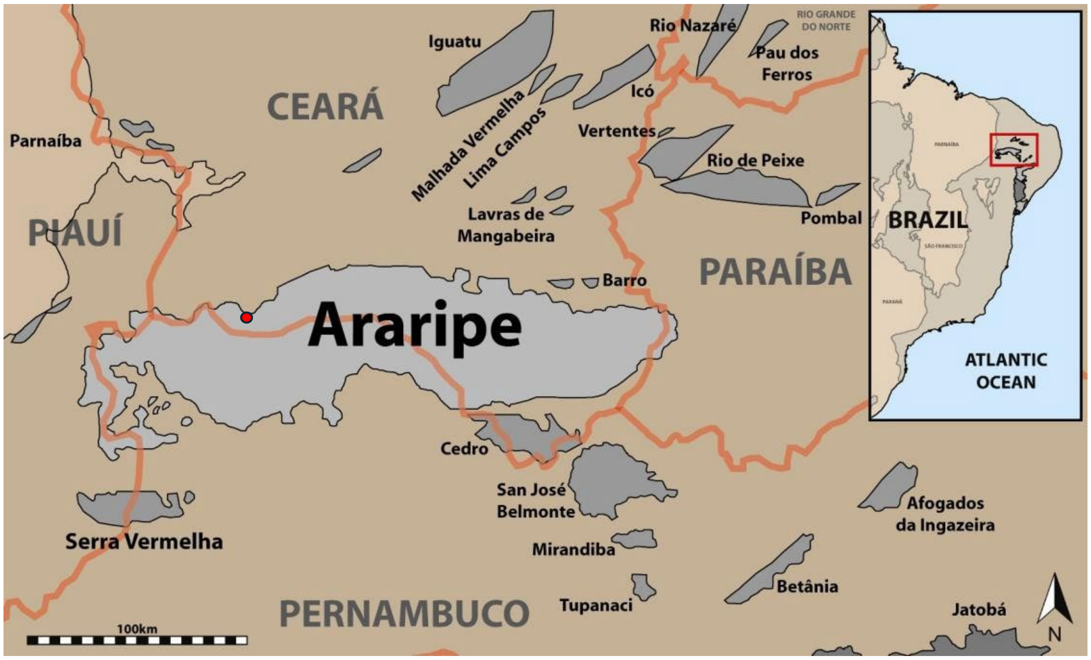

Fig. S1.
Map of the Araripe Basin, Brazil showing the locality (Sítio Baixa Grande) that produced the pterosaur specimen analysed herein.

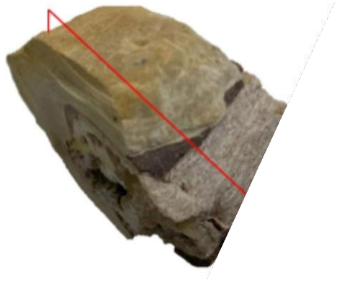

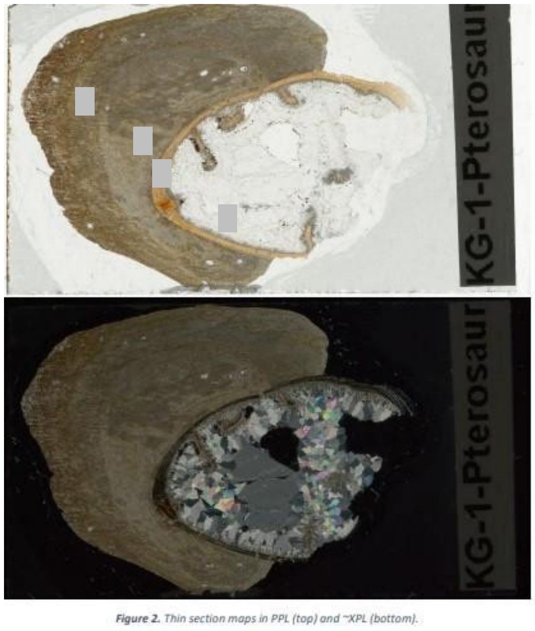

Fig. S2.
Sampling regions of specimen. Full slide dimensions of the thin section slides are 48 × 27 mm.

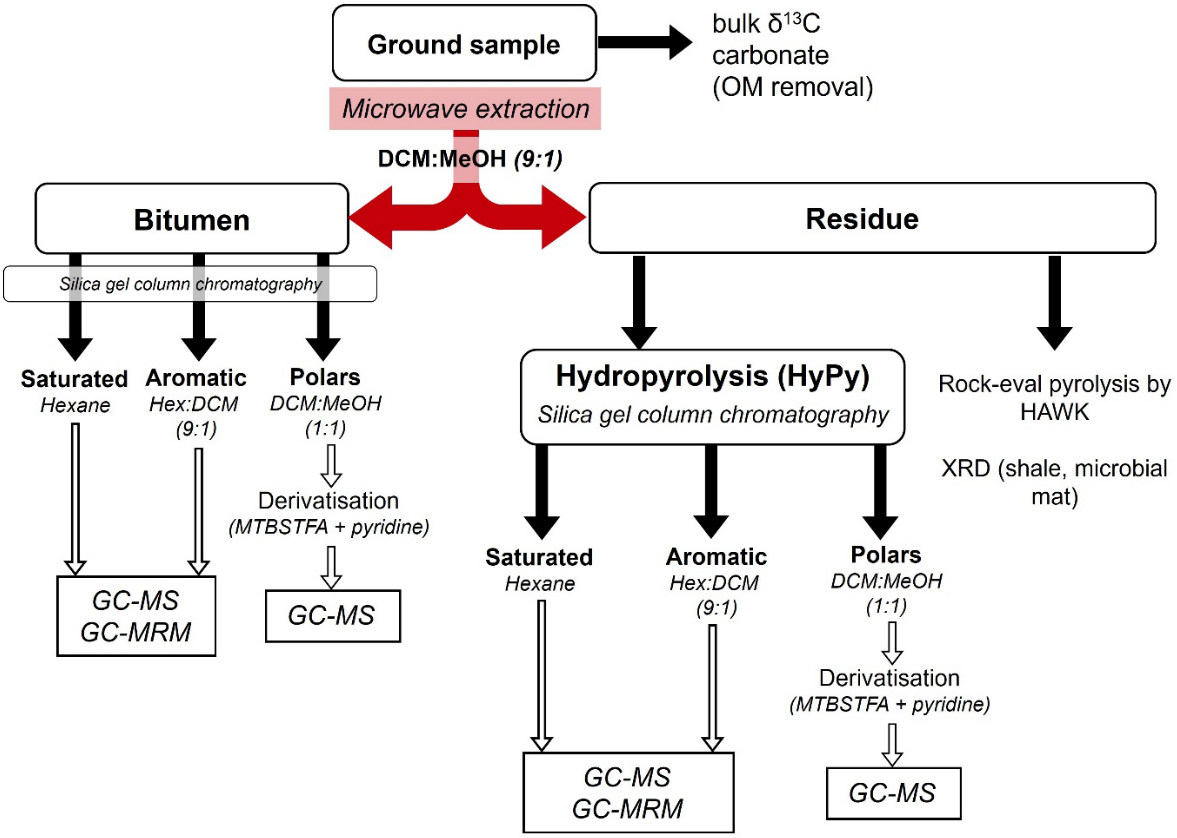

Fig. S3.
Analytical scheme showing the workflow employed herein for ground samples.

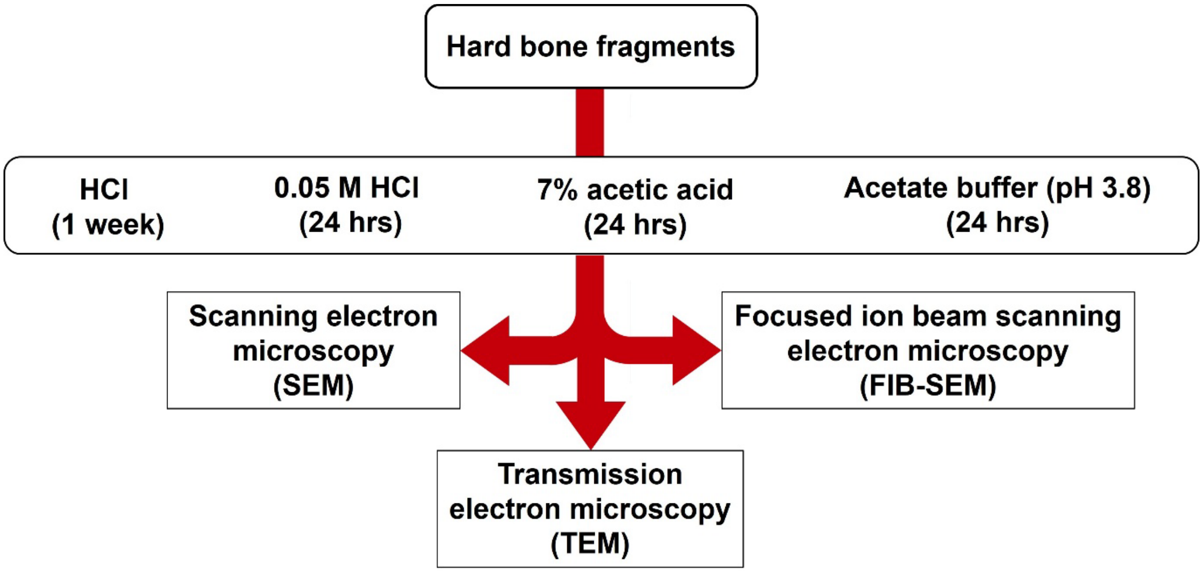

Fig. S4.

Analytical scheme showing the acid treatments workflow of hard bone subjected to SEM, EDS, FIB-SEM & TEM

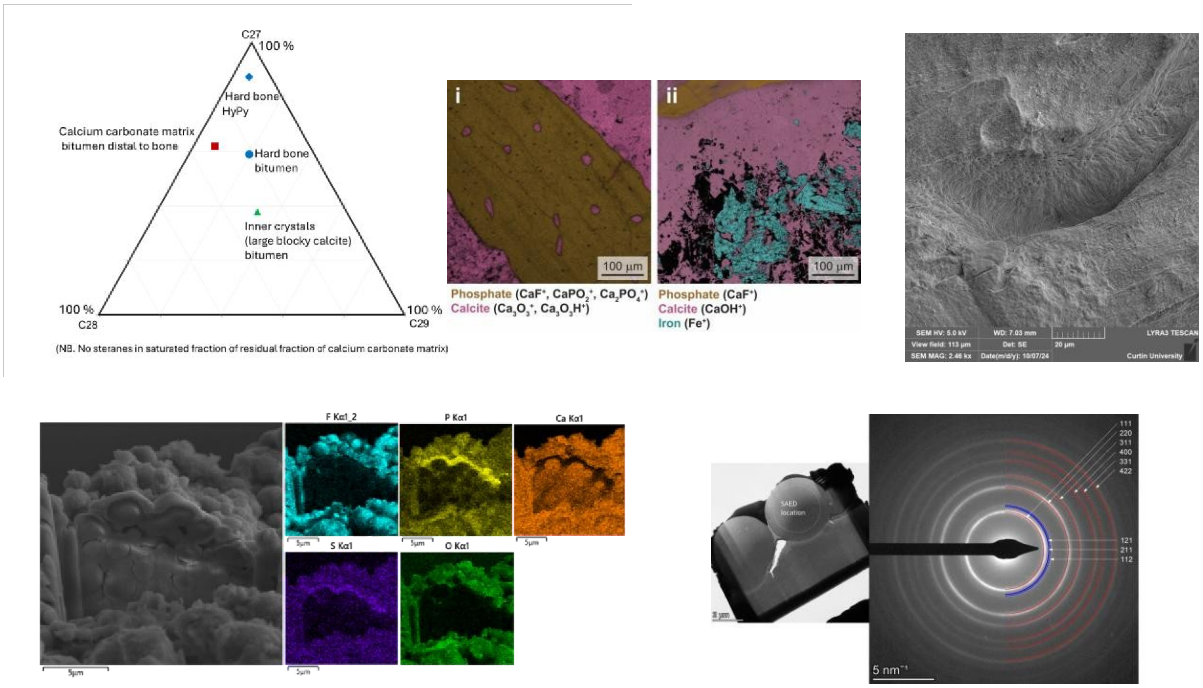

Fig. S5 The range of different techniques (molecular to micro and nano) utilized in our study to analyze the composition of the pterosaur specimen ranging from molecular biomarker geochemistry to ToF-SIMS, SEM-EDS, FIB-SEMS and TEM **(A.**Ternary diagram showing the % of C_{27}-C_{29} steranes in the saturated fraction of bitumen and residual fractions of hard bone (sample 1), inner calcite crystals (sample 2) and matrix (sample 4).

Fig. S5 (B. Overlaid ToF-SIMS secondary ion maps: **(i)** across the bone (uncoated thin section), showing the distributions of fluorapatite (brown – sum of CaF^+, $CaPO_2^+$ and $Ca_2PO_4^+$ ions; see fig. S11) and calcite (pink – sum of $Ca_3O_3^+$ and $Ca_3O_3H^+$ ions) at 500 × 500 µm; and **(ii)** adjacent to bone trusses, showing the presence of an iron-rich mineral phase (blue – Fe^+ ions), which also cooccurs with iron oxides and phosphates (see fig. S11 for individual secondary ion maps), overlaid with fluorapatite (brown – CaF^+ ions) and calcite (pink – $CaOH^+$ ions).

Fig. S5 (C. Scanning electron micrographs of the pterosaur bone taken after 24 hours of acetic acid treatment, revealing mineralized collagen fibers composed of fluorapatite.

Fig. S5 (D. FIB-SEM cross-section of fluorapatite spherulites on the pterosaur bone after 24 hours of hydrochloric acid treatment. The FIB-SEM image revealed their anuclear structure, while elemental mapping identified calcium (Ca), phosphorus (P), and sulfur (S) as the primary constituents (fig. S25)

Fig. S5 (E. Transmission Electron Microscopy showing nodules to be anuclear and semi-quant results of the EDS maps, appears to be consistent with a mixture of calcium fluorite and fluorapatite annotated SAED patterns, which show the expected ring positions for calcium fluorite and those of the most intense fluorapatite rings.

First Stage (Infill): Turbid microgranular (<10 µm) to granular (25 µm) calcite formed an inner rim around the bone cavity and the outer bone adjacent to the carbonate concretion. This calcite also lined the fine struts within the bone, as well as osteons within the specimen and phosphatized ostracods in the host matrix. This calcite rim, ranging from 25–100 µm thick, appears to be contiguous and contemporaneous with irregular or straight-sided fractures and veins of calcite that extend into or through the phosphatic bone material. These fractures do not crosscut the cements within the bone cavity, indicating that they formed post depositional but pre-cementation. Calcite veins appear contemporaneously with irregular overprinting of the phosphatic bone by calcite, which has ingressed from the outer surface. This suggests that calcite actively altered the structure of the bone at a later stage of fossilization. Larger osteocytes within the bone (~30–40 µm in size) are filled with granular calcite, further highlighting the diagenetic (post-burial) changes the bone has undergone.

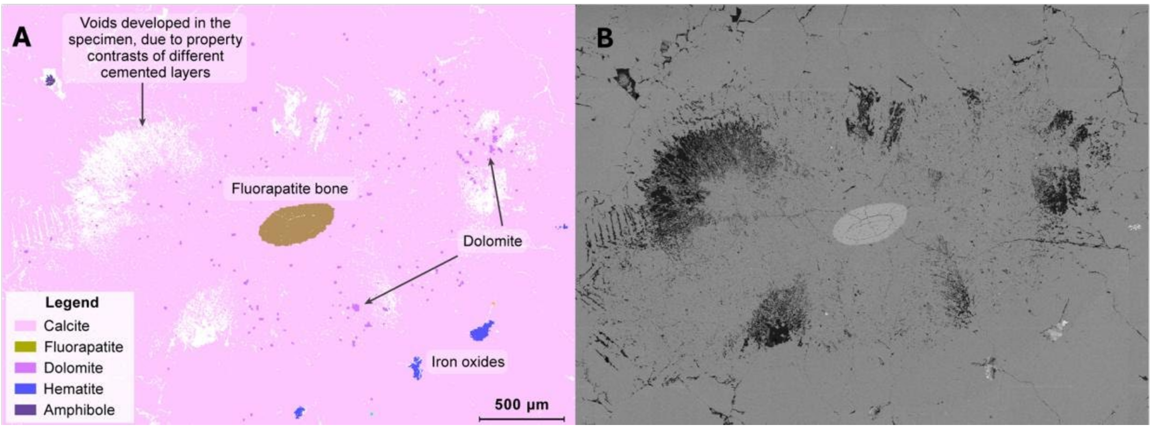

Fig. S6. TIMA map and BSE image.

(A. Automated mineralogical map with small piece of bone (fluorapatite). Dolomite grains in bladed calcite surrounding the bone, and voids due to property contrasts of cement layers and (**B.** Back scattered electron (BSE) image of the mineralogical map.

• Associated with vugs in euhedral calcite

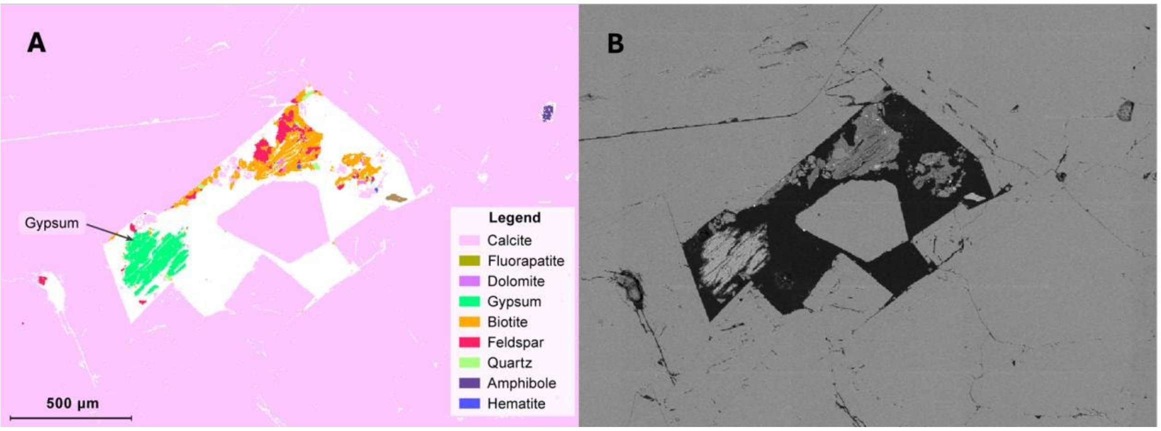

Fig. S7. TIMA map and BSE image.

(A. Automated mineralogical map of gypsum and other minerals in a vug within blocky calcite. and (**B.** BSE image of the mineralogical map.

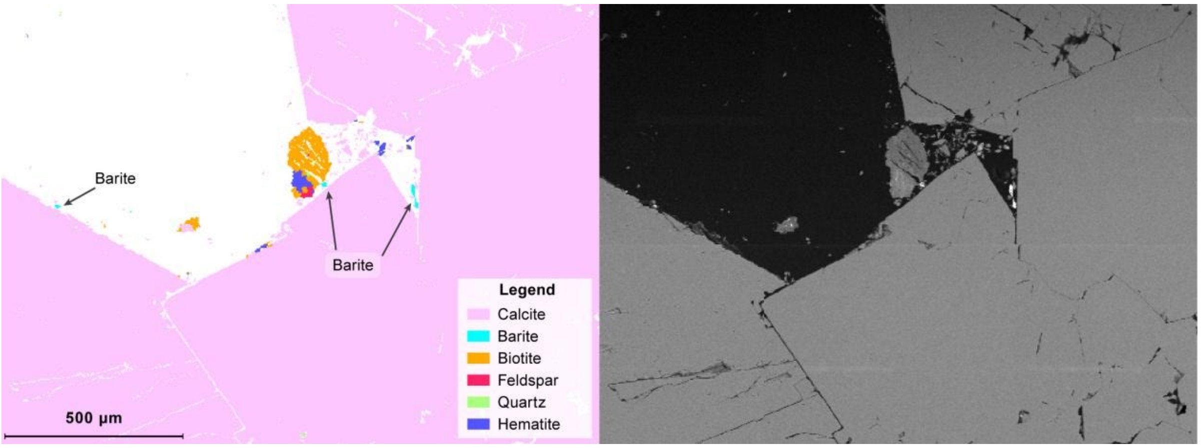

Fig. S8. TIMA map and BSE image.

(A) Automated mineralogical map of very fine barite and other mineral in a vug within blocky calcite and **(B)** BSE image of the mineralogical map.

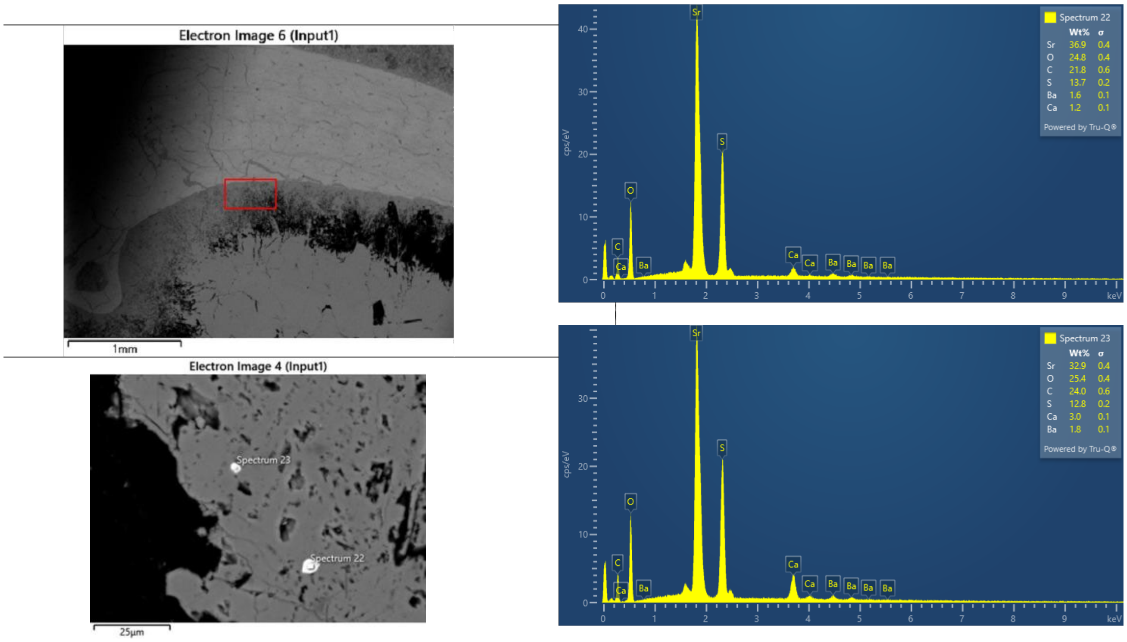

Fig. S9.

SEM-BSE image and associated EDS spectra for specific spot sites of phosphatic bone material in thin section (red inset shows spot location). Individual crystals of ~5-micron celestite are observed.

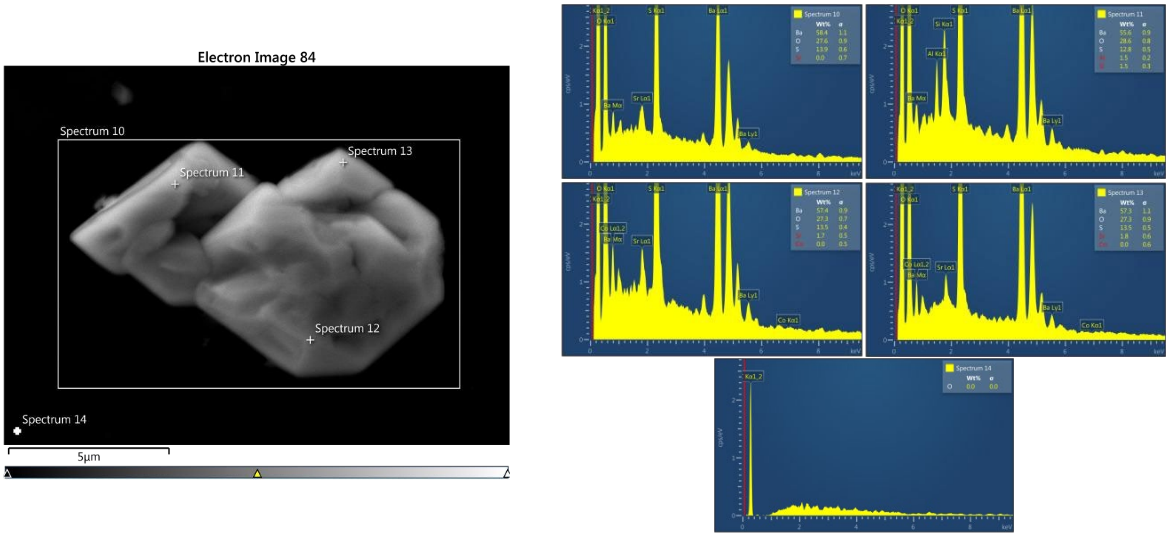

Fig. S10

SEM-BSE image and associated EDS spectra for specific spot sites of phosphatic bone material in thin section. Individual crystals of barite are observed.

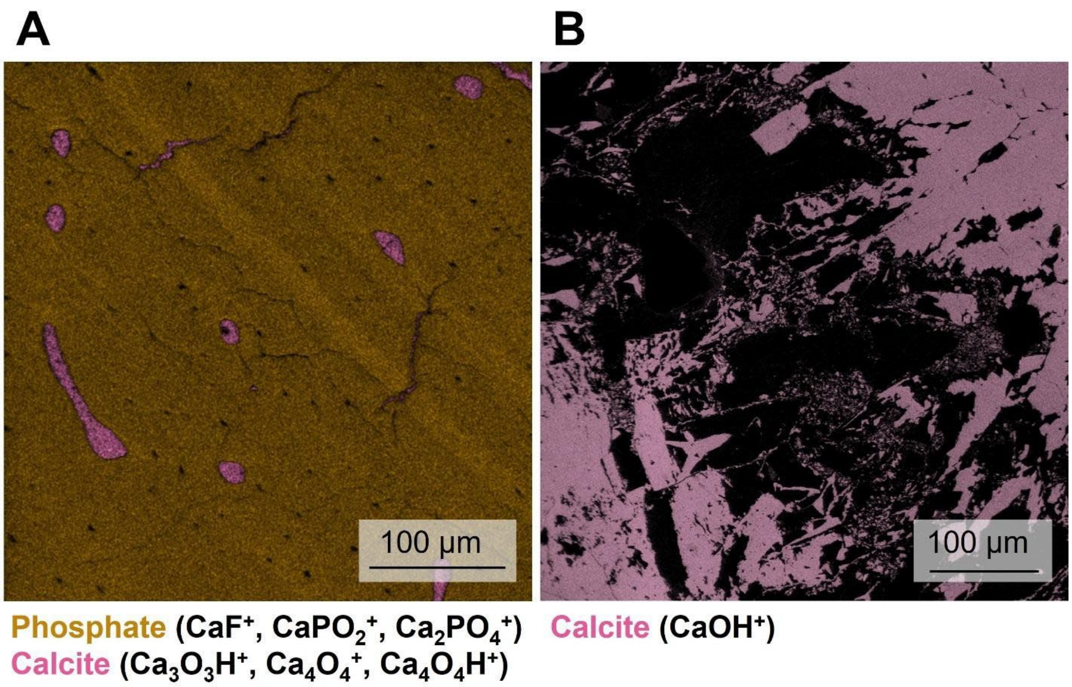

Fig. S11. Tof-SIMS secondary ion maps.

(A. Overlay of ToF-SIMS secondary ion maps showing the distributions of fluorapatite (in brown — sum of CaF^+, $CaPO_2^+$ and $Ca_2PO_4^+$ ions) and calcite (in pink — sum of $Ca_3O_3H^+$, $Ca_4O_4^+$ and $Ca_4O_4H^+$ ions) at high lateral resolution (500 × 500 µm) across the bone (thin section). The lamellar structure of the phosphatized bone can be observed where fluorapatite secondary ions are more abundant (brighter), as well as infill of osteocytes by calcite and (**B.** Secondary ions from calcite ($CaOH^+$) mapped at 500 × 500 µm across a void.

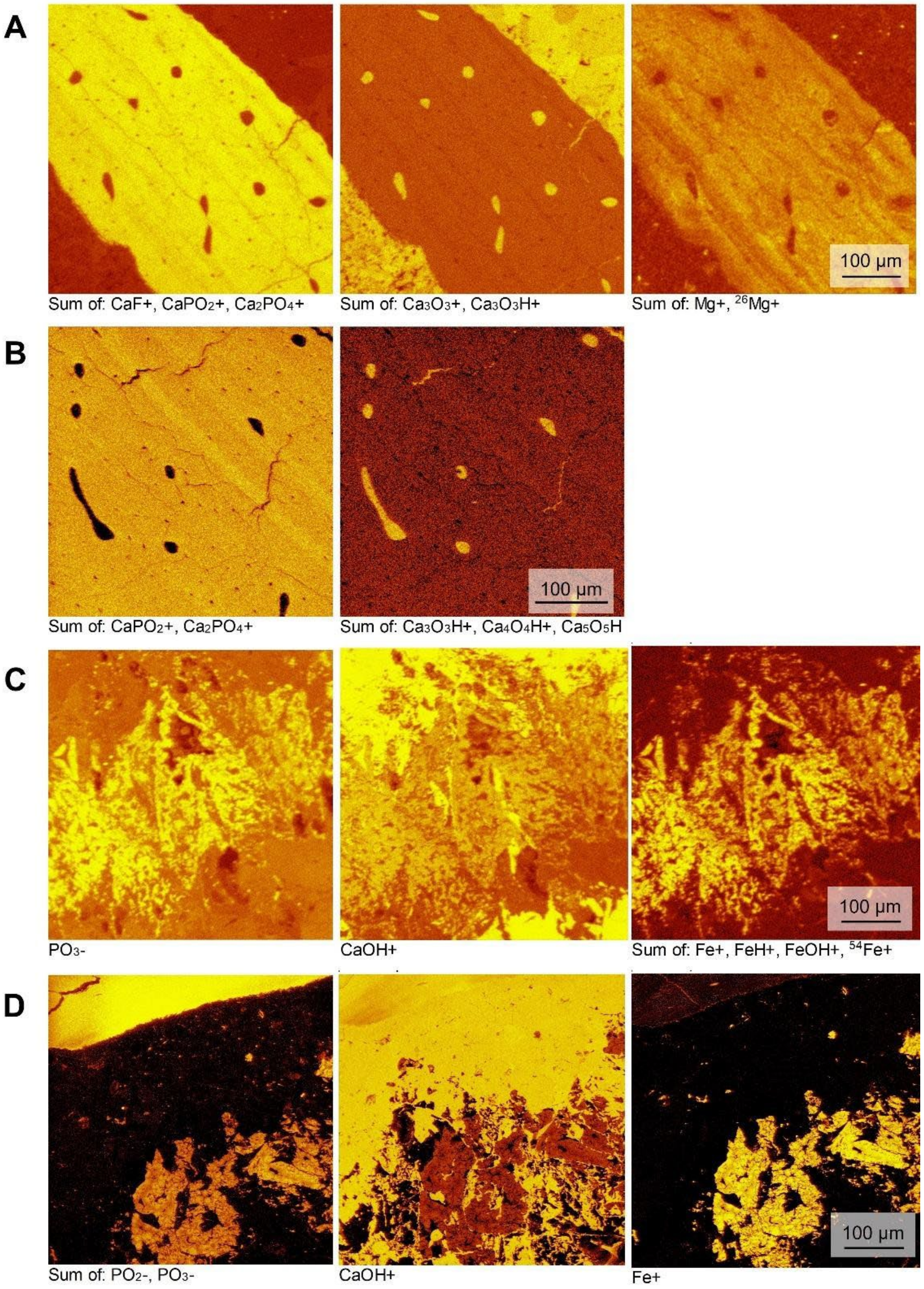

Fig. S12.

ToF-SIMS secondary ion maps across the bone region (**A** and **B)**, showing individual secondary ion abundance maps for overlays in fig. S5B and fig. S11A, respectively) and regions adjacent to the bone trusses (**C** and **D**). **C** and **D** depict iron oxide co-occurrence with phosphate.

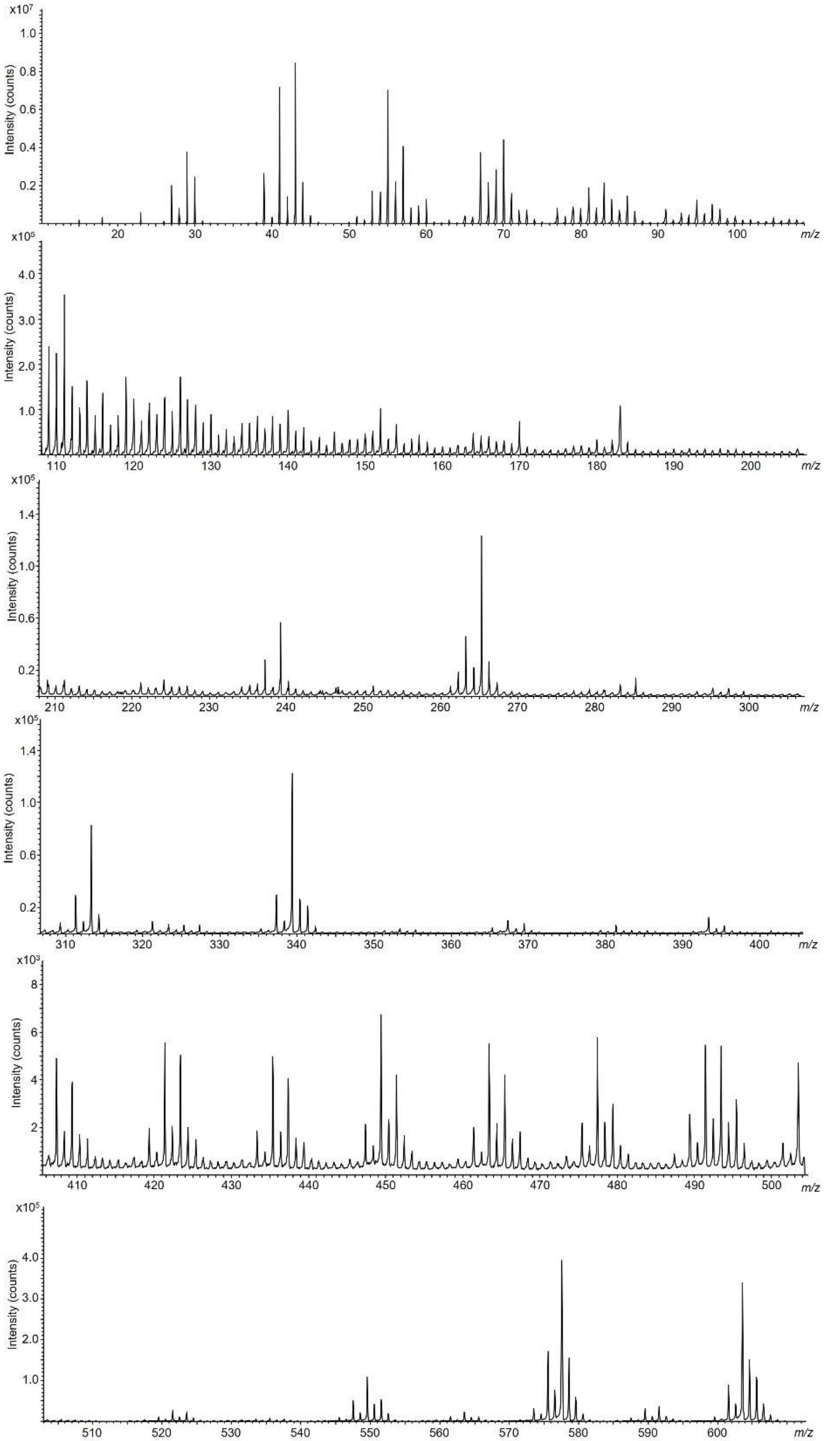

Fig. S13.

Positive ion ToF-SIMS spectra of pure collagen standard deposited on a silicon wafer, analyzed at high mass resolution. Ions were monitored up to approximately 1000 *m/z*, however no significant peaks were observed past 610 *m/z*.

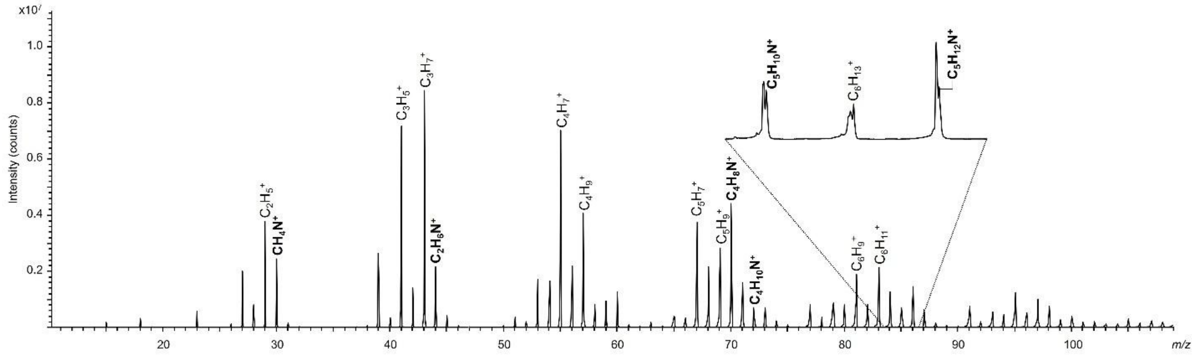

Fig. S14.

Low mass region (up to 110 *m/z*) of collagen standard in fig. S13, with major hydrocarbon peaks labelled alongside known amino acid fragments.

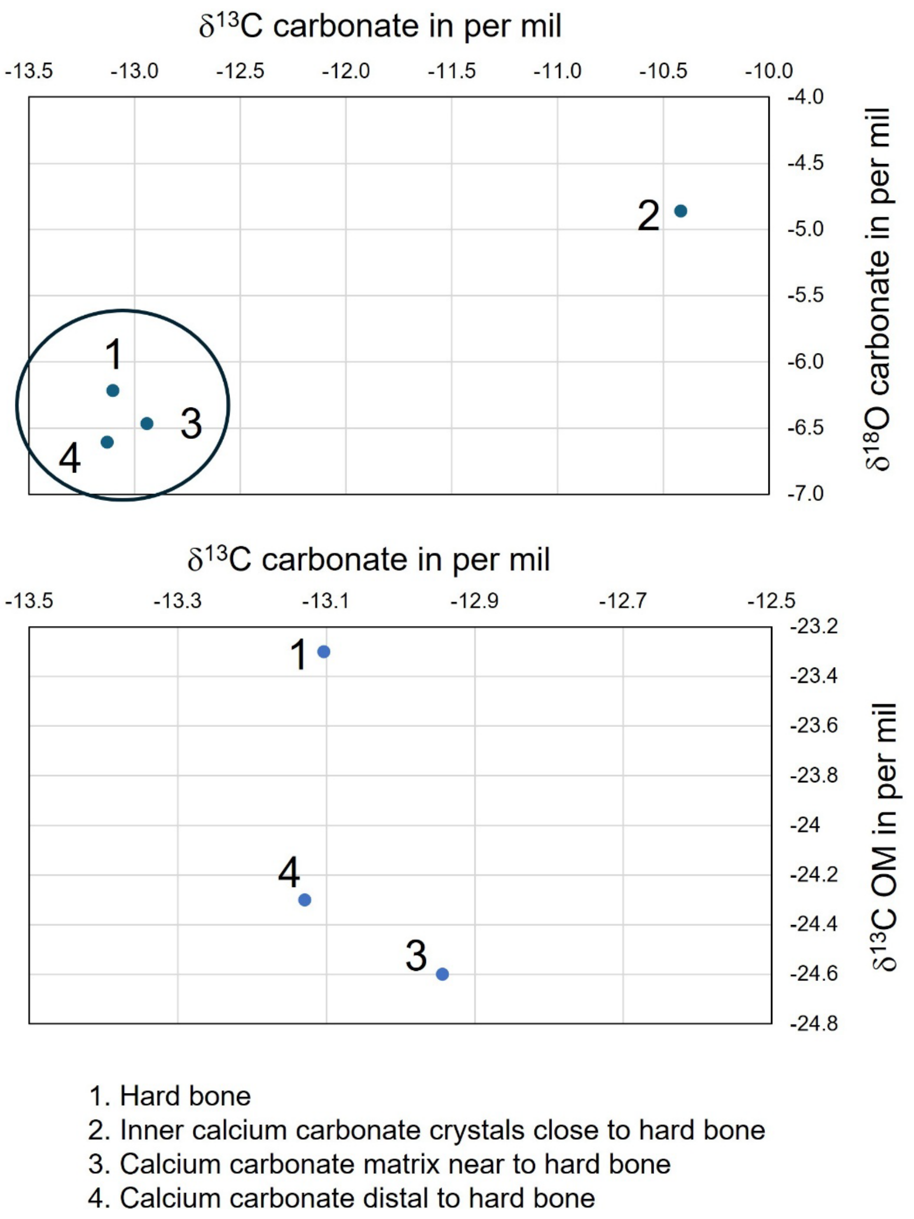

Fig. S15. $\delta^{13}C$ vs. $\delta^{18}O$ values of carbonates and $\delta^{13}C$ of carbonate vs. organic matter (see table S1 for raw data).

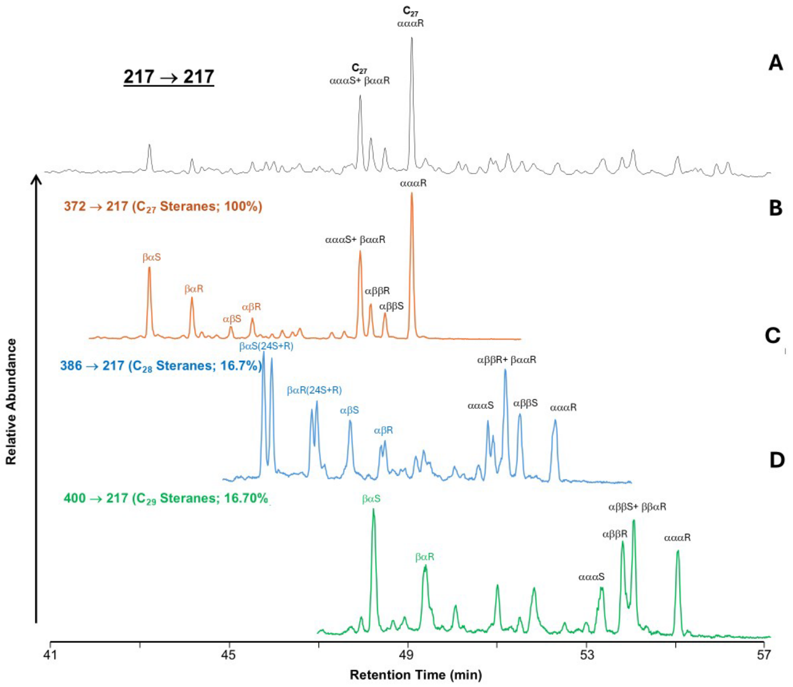

Fig. S16.

Sterane distributions highlighted by partial MRM transitions from GC-MSMS analysis of saturated fraction of hard bone (bitumen) (**A**. 217 → 217 of all C_{27} – C_{29} steranes; (**B**. 372 → 217 of C_{27} steranes; (**C**. 386 → 217 of C_{28} steranes; and (**D**. 386 → 217 of C_{29} steranes.

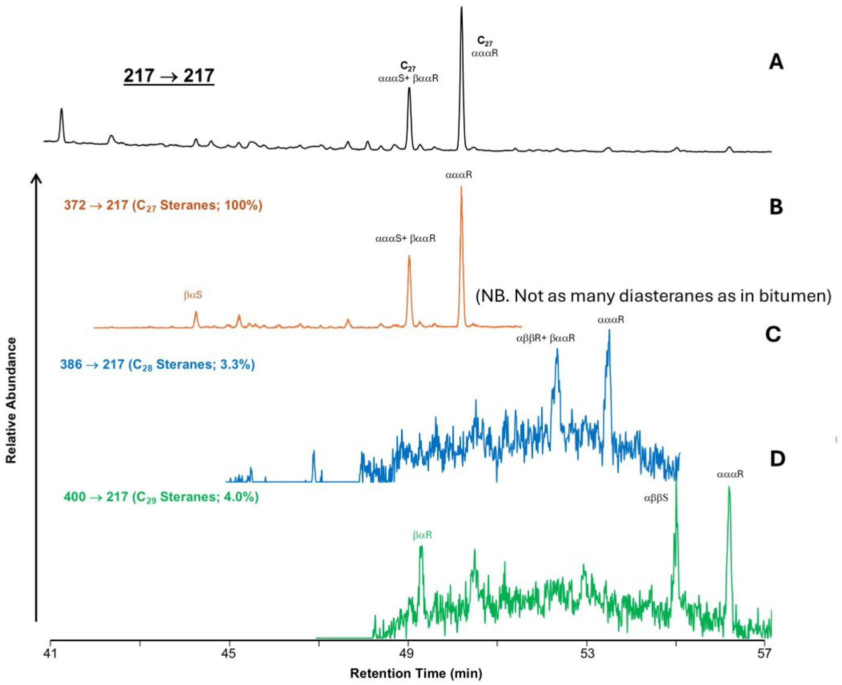

Fig. S17.

Sterane distributions highlighted by partial MRM transitions from GC-MSMS analysis of saturated fraction of hard bone (HyPy released kerogen) (**A**. 217 → 217 of all C_{27} – C_{29} steranes; (**B**. 372 → 217 of C_{27} steranes; (**C**. 386 → 217 of C_{28} steranes; and (**D**. 386 → 217 of C_{29} steranes.

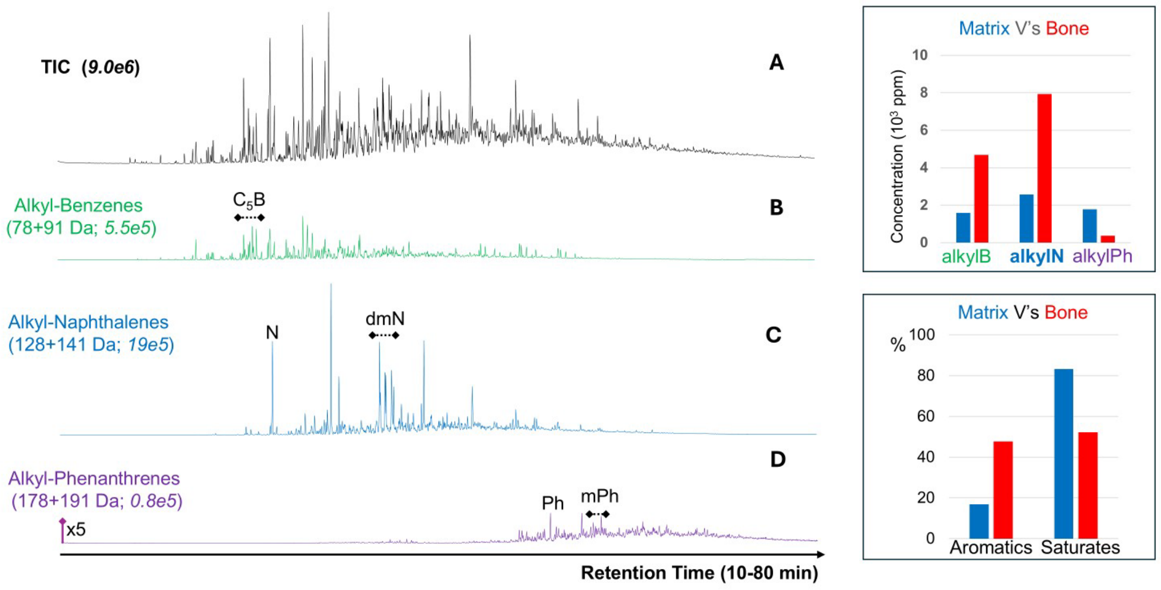

Fig. S18.

Aromatic hydrocarbon products highlighted by (**A**. total and (**B-D**. selected ion chromatograms from GC-MS analysis of aromatic fraction of hard bone (bitumen). Graphs at right compare matrix and bone product abundances.

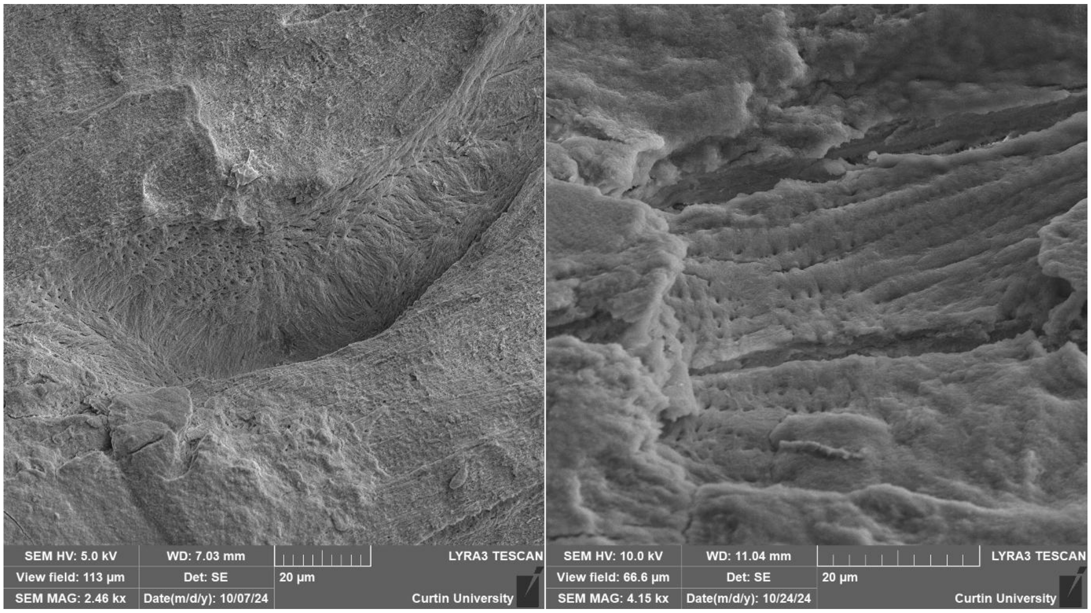

Fig. S19.

Scanning electron micrographs of the pterosaur bone after 24 hours of acetic acid treatment revealing mineralized collagen fibres composed of fluorapatite.

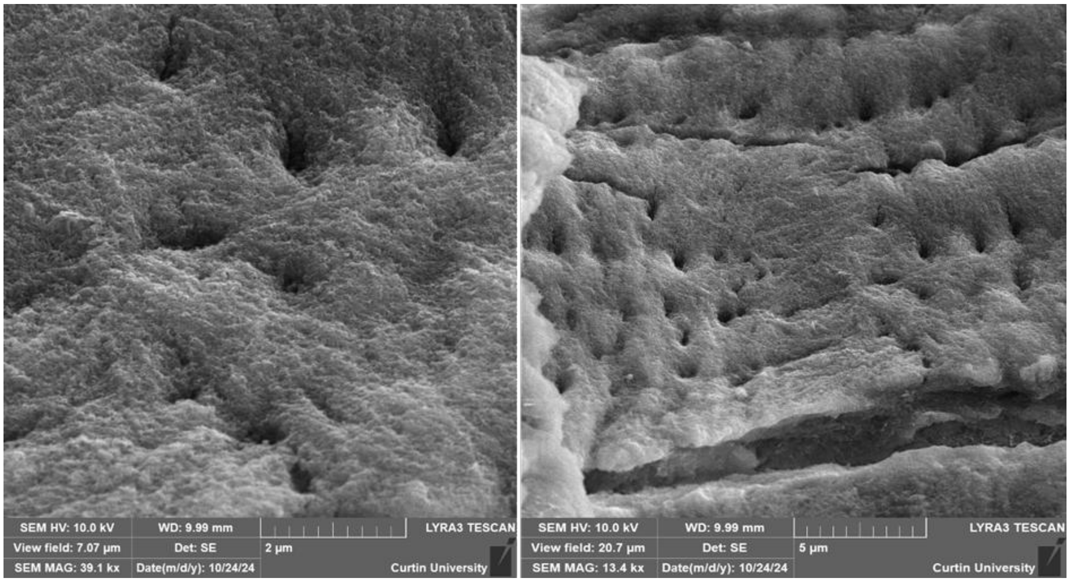

Fig. S20.

Higher magnification images of fig. S19, revealing mineralized collagen fibres and osteocyteassociated voids. These voids, initially filled with calcite, remained unfilled following a 24-hour acetic acid treatment.

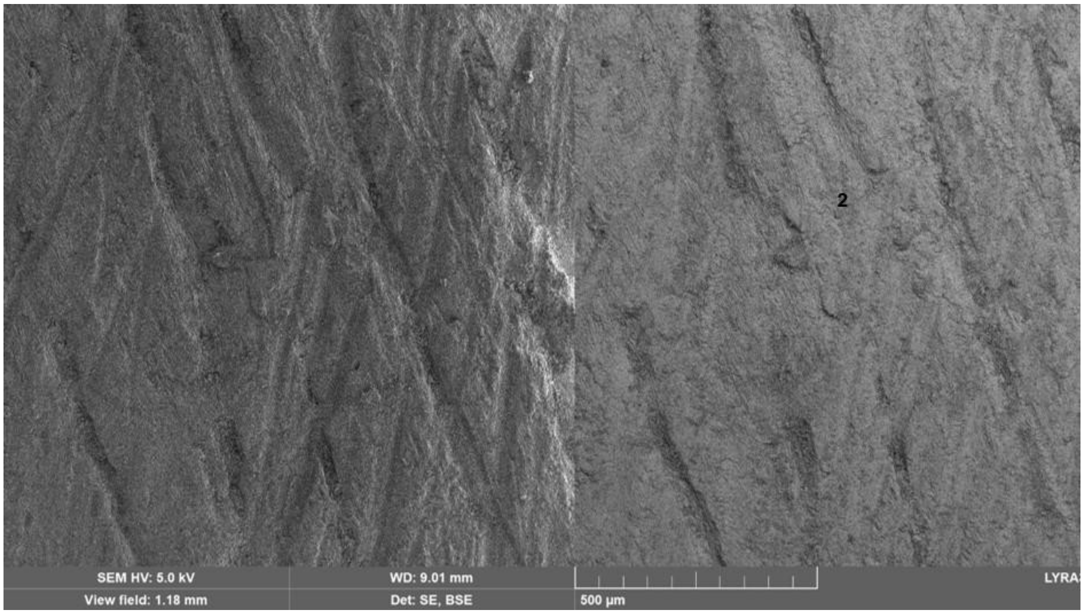

Fig. S21.

Low magnification secondary electron (SE) images of the pterosaur bone surface after 24 hours of acetic acid treatment showing a distinct crisscross pattern.

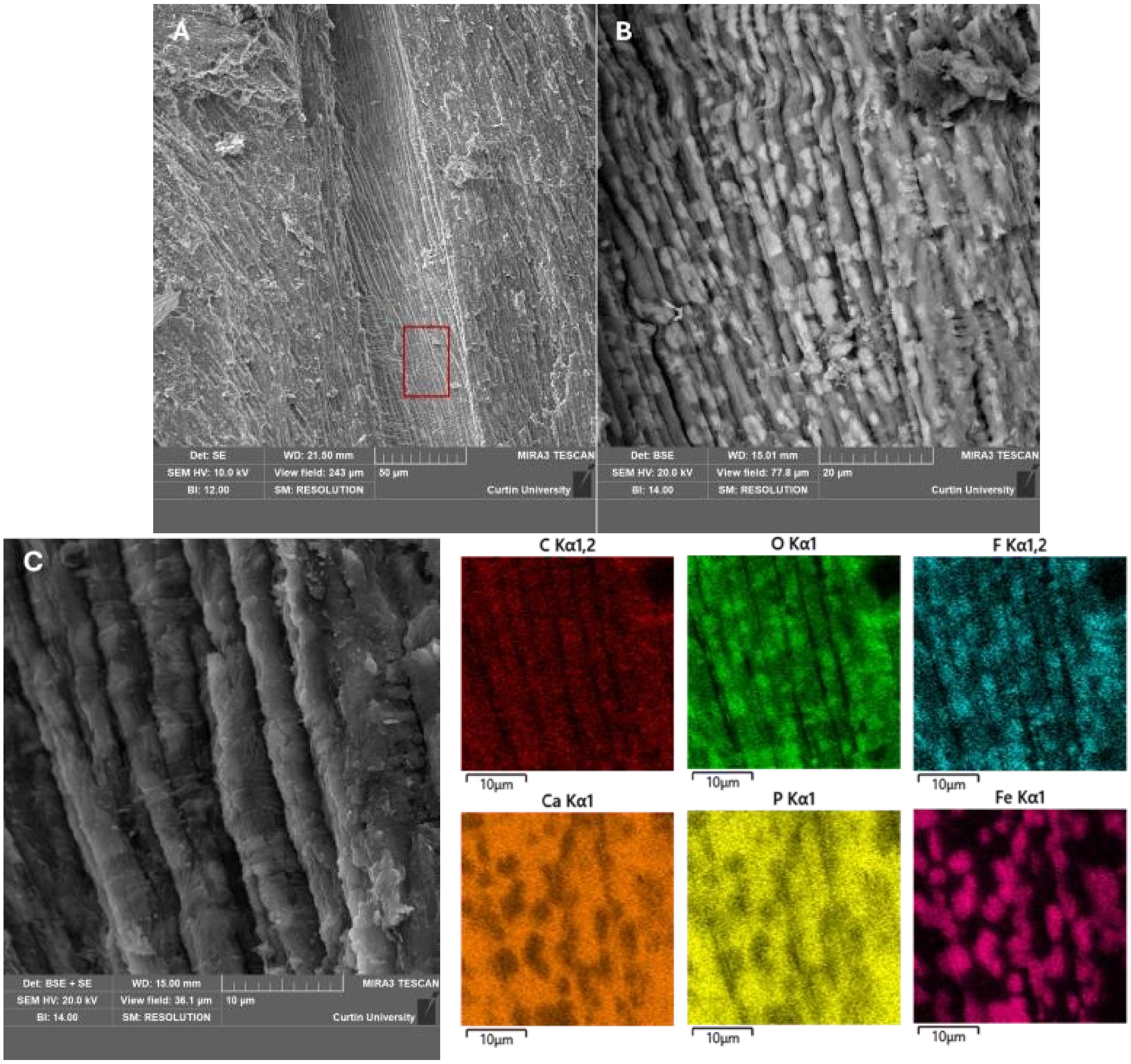

Fig. S22.

Scanning electron micrographs of the bone near the matrix surface (outer face) after 24 hours of acetic acid treatment. (**A.** SE overview image. (**B.** BSE image at higher magnification of **A**, revealing highly organized parallel collagen fibrils. Lighter regions within the fibrils indicate localized enrichment of higher atomic number elements. (**C**. SE image at increased magnification of (**A**. showing a mottled fibril texture and an average fibril diameter of 2–4 µm.

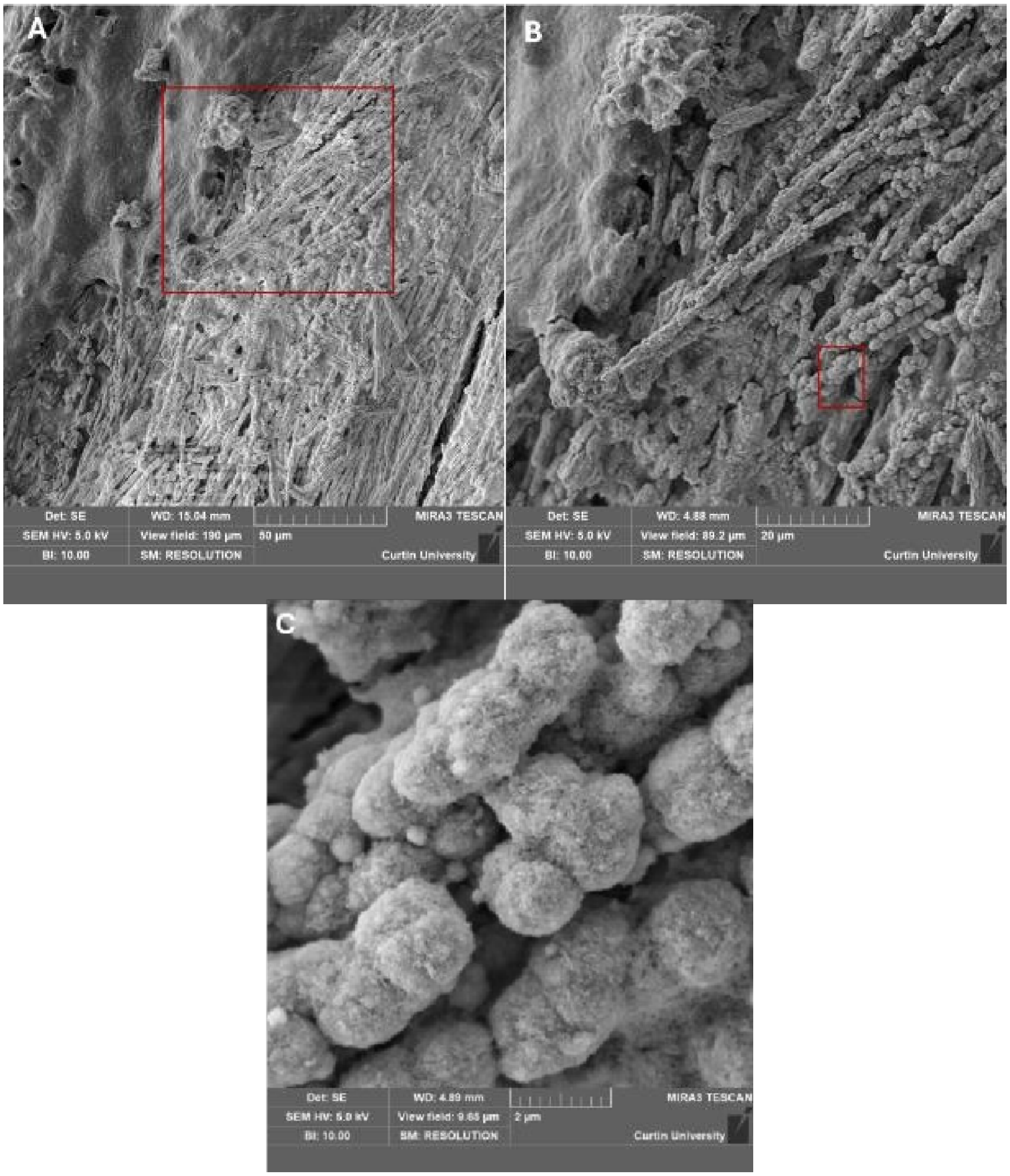

Fig. S23.

Scanning electron micrographs of the inner surface of pterosaur bone after 24 hours of hydrochloric acid treatment, revealing mineralized collagen fibres. (**A.** SE overview image. (**B.** Close-up SE image of **A**. showing fibres overgrown by spherical microcrystals of fluorapatite (spherulites). (**C**. Close-up SE image of **B** detailing the mottled texture of these spherulites, composed of aggregated fluorapatite nanocrystals.

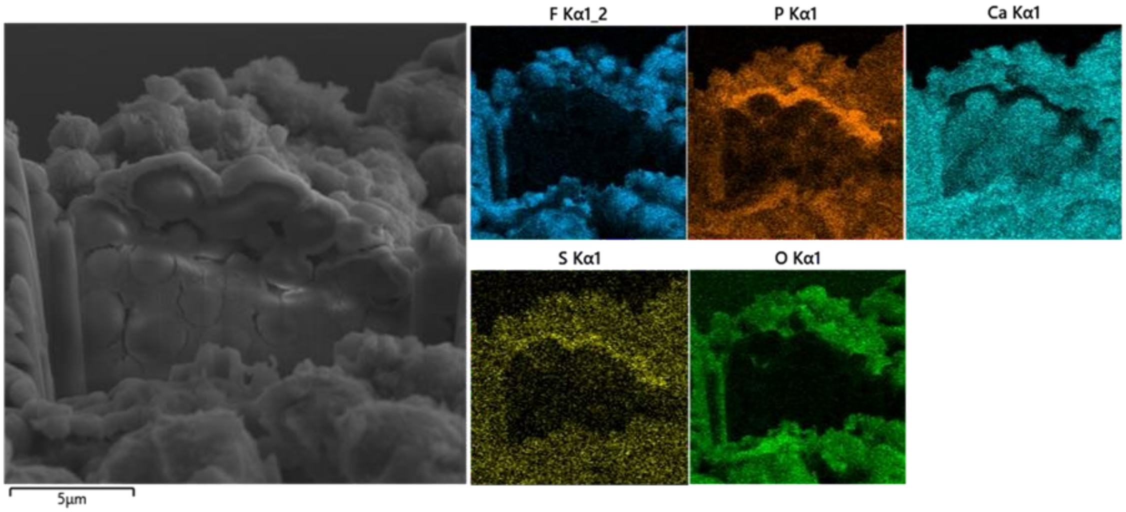

Fig. S24.

Energy-dispersive X-ray spectroscopy (EDS) analysis of a FIB-SEM cross-section of fluorapatite spherulites on the pterosaur bone after 24 hours of hydrochloric acid treatment. The FIB-SEM image revealed their anuclear structure, while elemental mapping identified calcium (Ca), phosphorus (P), and sulfur (S) as the primary constituents.

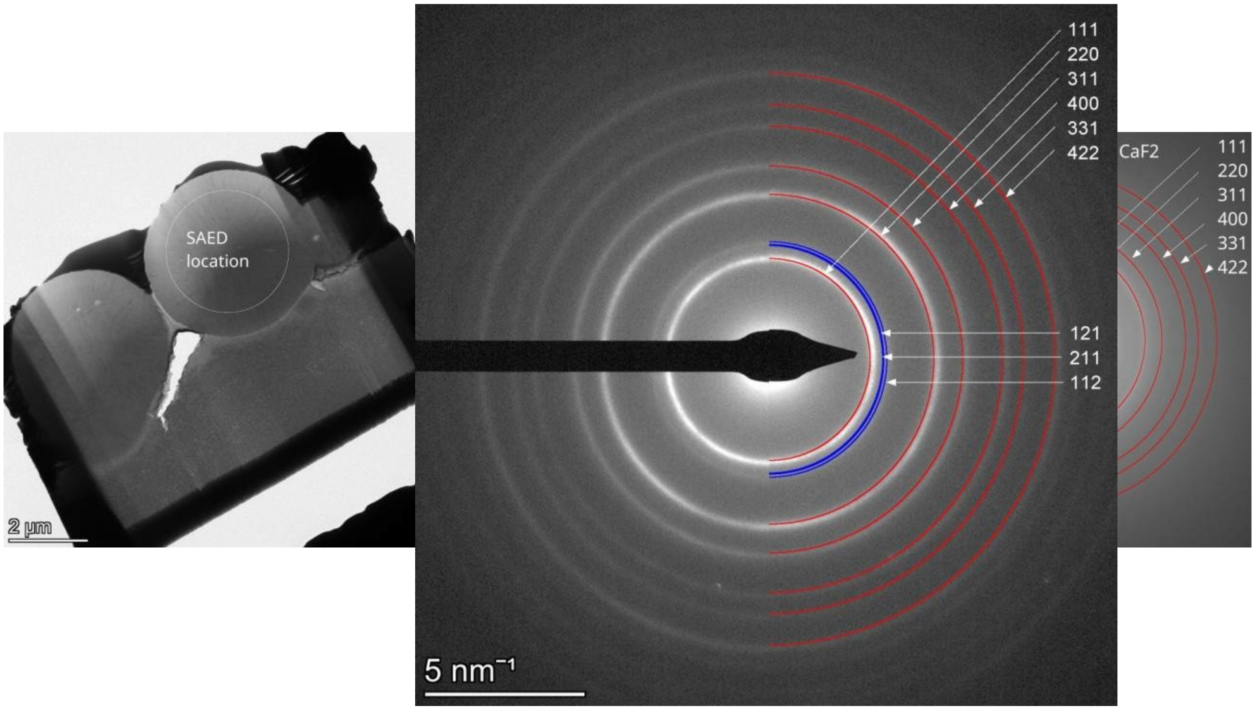

Fig. S25.

Lamellae prepared by FIB milling examined by transmission electron microscopy. Low and highresolution imaging was performed using TEM and STEM, which was coupled with EDS for elemental composition analysis. Diffraction patterns were taken for crystallographic characterization using selected area electron diffraction (SAED) identifying fluorapatite inside (blue) the nodules and calcium fluorite (red) around the outer edges.

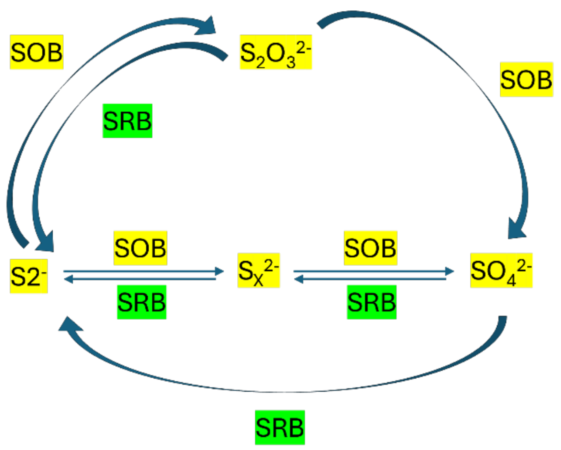

Equation 1 of simple sulfur cycle (schematic)

Table S1.

HAWK pyrolysis data, diasterane/sterane ratios, $\delta^{13}C$/ $\delta^{18}O$ of carbonates, $\delta^{13}C$ of organic matter, % of total sulfur (TS), $\delta^{34}S$ total sulfur and XRD (%).

Sample	TOC wt. %	HI mgHC/g TOC	OI mg CO_2/g TOC	Diasterane Sterane	$\delta^{13}C$ of carbonate ‰ (VPDB)	$\delta^{18}O$ of carbonate ‰ (VPDB)	$\delta^{13}C$ of TOC ‰ (VPDB)	TS wt %.	$\delta^{34}S$ TS ‰ (VCDT)	XRD %
1. Hard bone	1.21	38	298	0.75	-13.10 (0.04) -13.17*	-6.21 (0.04) -6.91*	-23.3 (0.04)	0.9	5.2	Fluorapatite 76.2 Calcite 23.8
2. Inner crystals near bone	0.94	9	89	0.72	-10.42 (0.03)	-4.86 (0.06)		0.4	8.5	
3. Calcium carbonate matrix near to hard bone	1.10	12	81	0.02	-12.94 (0.04)	-6.47 (0.06)	-24.6 (0.04)	-		
4. Calcium carbonate matrix distal to hard bone	0.69	11	191	0.02	-13.13 (0.06) -12.63*	-6.61 (0.06) -6.03*	-24.3 (0.04)	-		Calcite 88 Quartz 12
Outer sediment (Santana Formation, Romualdo Member)	Up to average 10.8	Up to average 810	40	0.25				-		

Total Organic Carbon (TOC), Hydrogen Indices (HI), Oxygen Indices (OI), Total Organic Carbon (TOC), Total sulfur (TS). * Additional measurements made at Kiel University. ** Stratigraphy of the Santana Group and Romualdo Formation was redefined by Neuman and Cabrera [18], however the additional data cited in Table S1 used the older stratigraphic divisions.

Table S2.
% of inorganic elements in samples and ppm of metals

Sample #	HCl leachate:	Ti [%]	Al [%]	Fe [%]	Ca [%]	Mg [%]	Na [%]	K [%]	P [%]	S [%]
1	Pterosaur hard brown bone	0.00	0.00	0.35	32.3	0.17	0.53	0.01	12.33	0.7
2	Pterosaur inner crystals near to bone	0.00	0.00	0.14	32.5	0.32	0.01	0.01	0.06	1.2
3	Pterosaur carbonate matrix near bone	0.00	0.05	0.07	34.6	0.24	0.03	0.04	0.13	0.1
4	Calcium carbonate matrix distal to the bone	0.00	0.04	0.07	33.2	0.22	0.02	0.03	0.23	0.1

Sample #	HCl leachate:	As [ppm] OES	Ba [ppm] OES	Cd [ppm] OES	Cr [ppm] OES	Cu [ppm] OES	Li [ppm] OES	Mn [ppm] OES	Mo [ppm] OES	Ni [ppm] OES	Pb [ppm] OES	Sr [ppm] OES	V [ppm] OES	Y [ppm] OES	Zn [ppm] OES
1	Pterosaur hard brown bone	11.5	29	3.2	5.1	116	4	389	92	5	26	1284	6	9	67
2	Pterosaur inner crystals near to bone	n.d.	19	0.2	n.d.	2	1	4691	1	0	n.d.	243	2	3	2
3	Pterosaur carbonate matrix near bone	n.d.	20	4.1	n.d.	10	2	1245	4	2	n.d.	266	11	7	5
4	Calcium carbonate matrix distal to the bone	n.d.	13	3.1	n.d.	11	2	1349	4	4	n.d.	254	14	12	7

Table S3. $\delta^{13}C$ of sterols and select aromatic compounds in samples

**Sample**	**$\delta^{13}C$ of C_{27} cholesterol** ‰ (VPDB) **Bitumen**	**$\delta_{13}C$ of C_{29} sterol** ‰ (VPDB) **Bitumen**	**$\delta^{13}C$ of Indane** ‰ (VPDB) **HyPy**	**$\delta^{13}C$ of 1,2,3,4 tetramethylbenzene** ‰ (VPDB) **HyPy**
1. Hard bone	-19.1 (0.4)[2]		-22.0 (0.29)[3]	-21.9 (0.37)[2]
4. Calcium carbonate matrix distal to hard bone	-	-29.2 (0.4)[2]		

Table S4

HAWK pyrolysis data for the additional Green River Formation and Posidonia Shale samples.

Sample	TOC wt. %	HI mgHC/g TOC	OI mg CO_2/g TOC
Green River Formation fossil vertebrae	0.9	464	90
Green River Formation matrix	1.6	686	33
Posidonia Shale fossil vertebrae	0.8	68	97
Posidonia Shale fossil ribs	1.3	198	79
Posidonia Shale dorsal concretion matrix	1.2	243	85
Posidonia Shale mid concretion matrix	1.1	166	54
Posidonia Shale ventral concretion matrix	1.2	287	46
Posidonia Shale host shale/rim	8.7	641	11

Total Organic Carbon (TOC), Hydrogen Indices (HI), Oxygen Indices (OI)

SI References

1. Love, G.D., Snape, C.E., Carr, A.D., and Houghton, R.C. (1995). Release of covalently-bound alkane biomarkers in high yields from kerogen via catalytic hydropyrolysis. Org. Geochem. *23*, 981-986. 10.1016/0146-6380(95)00075-5.
2. Grotheer, H., Robert, A.M., Greenwood, P.F., and Grice, K. (2015). Stability and hydrogenation of polycyclic aromatic hydrocarbons during hydropyrolysis (HyPy) – Relevance for high maturity organic matter. Org. Geochem. *86*, 45-54. 10.1016/j.orggeochem.2015.06.007.
3. Maende, A., Pepper, A., Jarvie, D.M., and Weldon, W.D. (2017). Advanced pyrolysis data and interpretation methods to identify unconventional reservoir sweet spots in fluid phase saturation and fluid properties (API gravity) from drill cuttings and cores. AAPG Search Discov. Art., 80596.
4. Jeppsson, L., Anehus, R., and Fredholm, D. (1999). The optimal acetate buffered acetic acid technique for extracting phosphatic fossils. J. Paleontol. *73*, 964-972. 10.1017/S0022336000040798.
5. Roeser, P., Böttcher, M., Liebezeit, A., Harms, U., Epp, L., Raschke, U., Schleheck, D., Schmiedinger, I., Anselmetti, F., Wessels, M., and Schwalb, A. (2024). Carbon diagenesis in dolomite- and calcite-bearing limnic sediments: A multi-phase stable isotope geochemical perspective on Lake Constance. held in Vienna, 14–19 April. pp. EGU24-17897.
6. Coplen, T.B., Brand, W.A., Gehre, M., Gröning, M., Meijer, H.A.J., Toman, B., and Verkouteren, R.M. (2006). New guidelines for δ13C measurements. Anal. Chem. *78*, 2439-2441. 10.1021/ac052027c.
7. Pollmann, T., Böttcher, M.E., and Giani, L. (2021). Young soils of a temperate barrier island under the impact of formation and resetting by tides and wind. CATENA *202*, 105275. 10.1016/j.catena.2021.105275.
8. Brand, W.A., and Coplen, T.B. (2012). Stable isotope deltas: tiny, yet robust signatures in nature. Isot. Environ. Health Stud. *48*, 393-409. 10.1080/10256016.2012.666977.
9. Mann, J.L., Vocke, R.D., and Kelly, W.R. (2009). Revised δ34S reference values for IAEA sulfur isotope reference materials S-2 and S-3. Rapid Commun. Mass Spectrom. *23*, 1116-1124. 10.1002/rcm.3977.
10. Zeng, L., Gätjen, J., Reinhardt, M., Böttcher, M.E., Reimer, A., Karius, V., Thiel, V., and Arp, G. (2023). Extremely 13C-enriched dolomite records interval of strong methanogenesis following a sulfate decline in the Miocene Ries impact crater lake. Geochim. Cosmochim. Acta *362*, 22-40. 10.1016/j.gca.2023.10.013.
11. Böttcher, M.E., Neubert, N., Escher, P., von Allmen, K., Samankassou, E., and Nägler, T.F. (2018). Multi-isotope (Ba, C, O) partitioning during experimental carbonatization of a hyper-alkaline solution. Geochemistry *78*, 241-247. 10.1016/j.chemer.2018.01.001.
12. Flügel, E. (2004). Microfacies of Carbonate Rocks: Analysis, Interpretation and Application (Springer). 10.1007/978-3-662-08726-8.
13. Holman, A.I., Poropat, S.F., Greenwood, P.F., Bhandari, R., Tripp, M., Hopper, P., Schimmelmann, A., Brosnan, L., Rickard, W.D.A., Wolkenstein, K., and Grice, K. (2024). Significance of lignin and fungal markers in the Devonian (407 Ma) Rhynie Chert. Geobiology *22*, e12616. 10.1111/gbi.12616.
14. Tripp, M., Wiemann, J., Brosnan, L., Rickard, W.D.A., Vajda, V., Böttcher, M.E., Greenwood, P.F., and Grice, K. (2025). Mineralization controls informative biomarker preservation associated with soft part fossilization in deep time. Geobiology *23*, e70030. 10.1111/gbi.70030.
15. Toporski, J., and Steele, A. (2004). Characterization of purified biomarker compounds using time of flight-secondary ion mass spectrometry (ToF-SIMS). Org. Geochem. *35*, 793-811. 10.1016/j.orggeochem.2004.03.006.
16. Wagner, M.S., and Castner, D.G. (2001). Characterization of adsorbed protein films by time-of-flight secondary ion mass spectrometry with principal component analysis. Langmuir *17*, 4649-4660. 10.1021/la001209t.
17. Siljeström, S., Lausmaa, J., Sjövall, P., Broman, C., Thiel, V., and Hode, T. (2010). Analysis of hopanes and steranes in single oil-bearing fluid inclusions using time-of-flight secondary ion mass spectrometry (ToF-SIMS). Geobiology *8*, 37-44. 10.1111/j.14724669.2009.00223.x.

18. Neumann, V.H., and Cabrera, L. (1999). Una nueva propuesta estratigrafica para la tectonosecuencia post-rifte de la cuenca de Araripe, noreste de Brasil. In Boletim do 5° Simpósio sobre o Cretáceo do Brasil, (UNESP São Paulo State University), pp. 279-285.
